# Proposed refined diagnostic criteria and classification of eosinophil disorders and related syndromes

**DOI:** 10.1111/all.15544

**Published:** 2022-10-19

**Authors:** Peter Valent, Amy D. Klion, Florence Roufosse, Dagmar Simon, Georgia Metzgeroth, Kristin M. Leiferman, Juliana Schwaab, Joseph H. Butterfield, Wolfgang R. Sperr, Karl Sotlar, Peter Vandenberghe, Gregor Hoermann, Torsten Haferlach, Richard Moriggl, Tracy I. George, Cem Akin, Bruce S. Bochner, Jason Gotlib, Andreas Reiter, Hans‐Peter Horny, Michel Arock, Hans‐Uwe Simon, Gerald J. Gleich

**Affiliations:** ^1^ Department of Internal Medicine I, Division of Hematology & Hemostaseology Medical University of Vienna Vienna Austria; ^2^ Ludwig Boltzmann Institute for Hematology and Oncology Medical University of Vienna Vienna Austria; ^3^ Human Eosinophil Section, Laboratory of Parasitic Diseases NIH/NIAID Bethesda MD USA; ^4^ Department of Internal Medicine Erasme Hospital, Université Libre de Bruxelles Brussels Belgium; ^5^ Department of Dermatology, Inselspital Bern University Hospital, University of Bern Bern Switzerland; ^6^ Department of Hematology and Oncology University Hospital Mannheim ‐ Heidelberg University Mannheim Germany; ^7^ Department of Dermatology University of Utah Health Salt Lake City UT USA; ^8^ Division of Allergic Diseases Mayo Clinic Rochester MN USA; ^9^ Institute of Pathology University Hospital Salzburg, Paracelsus Medical University Salzburg Austria; ^10^ Division of Hematology University Hospital Leuven and Department of Human Genetics, KU Leuven Leuven Belgium; ^11^ MLL Munich Leukemia Laboratory Munich Germany; ^12^ Institute of Animal Breeding and Genetics University of Veterinary Medicine Vienna Austria; ^13^ Department of Pathology University of Utah Salt Lake City UT USA; ^14^ Division of Allergy and Clinical Immunology University of Michigan Ann Arbor MI USA; ^15^ Northwestern University Feinberg School of Medicine Division of Allergy and Immunology Chicago IL USA; ^16^ Stanford Cancer Institute/Stanford University School of Medicine Stanford CA USA; ^17^ Institute of Pathology Ludwig Maximilian University Munich (LMU) Munich Germany; ^18^ Department of Hematological Biology Pitié‐Salpêtrière Hospital, Pierre et Marie Curie University (UPMC) Paris France; ^19^ Institute of Pharmacology University of Bern Bern Switzerland; ^20^ Institute of Biochemistry Brandenburg Medical School Neuruppin Germany; ^21^ Departments of Dermatology and Medicine University of Utah Health Salt Lake City UT USA

**Keywords:** classification, diagnostic criteria, eosinophilic leukemia, hypereosinophilic syndrome, personalized medicine

## Abstract

Eosinophilia and eosinophil activation are recurrent features in various reactive states and certain hematologic malignancies. In patients with hypereosinophilia (HE), HE‐induced organ damage is often encountered and may lead to the diagnosis of a hypereosinophilic syndrome (HES). A number of known mechanisms and etiologies contribute to the development of HE and HES. Based on these etiologies and the origin of eosinophils, HE and HES are divided into primary forms where eosinophils are clonal cells, reactive forms where an underlying reactive or neoplastic condition is detected and eosinophils are considered to be “non‐clonal” cells, and idiopathic HE and HES in which neither a clonal nor a reactive underlying pathology is detected. Since 2012, this classification and the related criteria have been widely accepted and regarded as standard. However, during the past few years, new developments in the field and an increasing number of markers and targets have created a need to update these criteria and the classification of HE and HES. To address this challenge, a Working Conference on eosinophil disorders was organized in 2021. In this conference, a panel of experts representing the relevant fields, including allergy, dermatology, hematology, immunology, laboratory medicine, and pathology, met and discussed new markers and concepts as well as refinements in definitions, criteria and classifications of HE and HES. The outcomes of this conference are presented in this article and should assist in the diagnosis and management of patients with HE and HES in daily practice and in the preparation and conduct of clinical trials.

**Abbreviations**
AEC: Absolute eosinophil count
AEL: Acute eosinophilic leukemia(s)
AML: Acute myeloid leukemia
ANCA: Anti‐neutrophil cytoplasmic antibodies
BCR: Breakpoint cluster region
BM: Bone marrow
CEL: Chronic eosinophilic leukemia(s)
CML: Chronic myeloid leukemia
eGPA: Eosinophilic granulomatosis with polyangiitis
eMBP1: Eosinophil (granule) major basic protein 1
EPX: Eosinophil peroxidase
FGFR: Fibroblast growth factor receptor
FISH: Fluorescence in situ hybridization
GM‐CSF: Granulocyte/macrophage colony‐stimulating factor
HE: Hypereosinophilia
HES: Hypereosinophilic syndrome
ICOG‐EO: International cooperative study group on eosinophil disorders
IgG4‐RD: Immunoglobulin‐G4‐related disease
IL: Interleukin
JAK: Janus kinase
MBP: Major basic protein
MDS: Myelodysplastic syndrome(s)
MLN‐TK: Myeloid/lymphoid neoplasms with eosinophilia and tyrosine kinase gene fusions
MPN: Myeloproliferative neoplasm(s)
NGS: Next generation sequencing
NHL: Non Hodgkin lymphoma
PB: Peripheral blood
PCR: Polymerase chain reaction
PDGF: Platelet‐derived growth factor
PDGFR: Platelet‐derived growth factor receptor
SM: Systemic mastocytosis
TcR: T‐cell receptor
TGF: Transforming growth factor
VAF: Variant allele frequency
WHO: World Health Organization

## 1 INTRODUCTION

Eosinophilia is observed in a number of inflammatory and other reactive conditions and related disease states, as well as in several hematologic malignancies.^1^, ^2^, ^3^, ^4^, ^5^, ^6^, ^7^, ^8^, ^9^, ^10^, ^11^, ^12^ In reactive states, eosinophilia is usually a non‐neoplastic process triggered by eosinophil‐targeting cytokines, such as interleukin‐3 (IL‐3) or IL‐5.^1^, ^2^, ^3^, ^4^, ^5^, ^6^, ^12^, ^13^ In contrast, in stem cell‐derived and myeloid neoplasms, eosinophils usually derive from the malignant clone.^6^, ^7^, ^8^, ^9^, ^10^, ^11^, ^13^, ^14^, ^15^, ^16^ Reactive eosinophilia may be transient or episodic (recurrent) but may also persist. In contrast, clonal (neoplastic) eosinophilia is always a persistent condition unless the disease progresses to an acute leukemia or specific anti‐neoplastic therapy is introduced. Blood eosinophilia is defined by an absolute eosinophil count (AEC) of more than 0.5 × 10^9^/L, whereas hypereosinophilia (HE) requires an AEC of ≥1.5 × 10^9^/L.^4^, ^5^, ^6^, ^7^, ^14^, ^15^, ^16^

In patients with persistent (or recurrent) hypereosinophilia (HE), tissue infiltration by eosinophils and release of eosinophil‐derived mediators and cytotoxic proteins may result in clinically relevant organ damage and thus a hypereosinophilic syndrome (HES).^1^, ^2^, ^3^, ^4^, ^5^, ^6^, ^12^, ^13^, ^14^, ^15^ In other patients, HE is persistent but does not lead to detectable organ damage. These patients must be examined carefully and repeatedly for the development of HE‐related manifestations during follow‐up.^14^, ^15^, ^16^

Several neoplastic conditions are associated with eosinophilia.^6^, ^7^, ^8^, ^9^, ^10^, ^11^, ^14^, ^15^, ^16^ Myeloid neoplasms frequently accompanied by eosinophilia include eosinophilic leukemias, chronic myeloid leukemia (CML), other myeloproliferative neoplasms (MPN), distinct variants of acute myeloid leukemia (AML), rare forms of myelodysplastic syndromes (MDS), some MDS/MPN overlap disorders, and a subset of patients with (advanced) systemic mastocytosis (SM).^6^, ^7^, ^8^, ^9^, ^10^, ^11^, ^14^, ^15^, ^16^ These diagnoses must be considered in cases of unexplained eosinophilia, especially when signs of dysplasia and/or myeloproliferation are present. In such patients, a thorough hematologic work‐up, including bone marrow (BM) cytology, histopathology, immunohistochemistry, cytogenetics, molecular analyses, and staging of potentially affected organ systems, are warranted.^6^, ^7^, ^8^, ^9^, ^10^, ^11^, ^14^, ^15^, ^16^ In all HE‐related disorders, including hematologic neoplasms with HE, eosinophil‐related organ damage may occur, especially when treatment is delayed or the disease is treatment‐resistant. In myeloid/lymphoid neoplasms with eosinophilia and rearrangements in the platelet‐derived growth factor receptor genes (*PDGFRs*), patients require early treatment with specific drugs to minimize the risk of (i) hematologic progression and (ii) occurrence of thromboembolic or fibrotic complications or other manifestations of HES. Imatinib is an effective therapy that leads to complete remission in nearly all of these patients.^17^, ^18^, ^19^, ^20^, ^21^

During the past two decades, several classifications of eosinophil disorders have been proposed.^6^, ^7^, ^8^, ^9^, ^10^, ^11^, ^14^, ^15^, ^16^, ^22^, ^23^, ^24^, ^25^ In 2011, a multidisciplinary international cooperative working group (ICOG‐EO) was convened to establish diagnostic criteria and a global classification of eosinophil disorders and related syndromes.^15^ This classification, published in 2012, is widely used, as it is easily applicable in daily practice and includes disease‐related markers and aspects from various fields of medicine, including allergy, hematology, immunology, pathology, and laboratory medicine.^15^, ^16^, ^23^

Over the past 10 years, additional markers and disease‐triggering mechanisms have been identified, and novel concepts concerning the diagnosis, prognosis, and therapy of eosinophil disorders have been developed. The World Health Organization (WHO) has provided updated criteria and classifications for hematopoietic neoplasms accompanied by eosinophilia in 2017 and 2022.^8^, ^9^, ^10^, ^11^ In complement to these updates, the ICOG‐EO organized the Year 2021 Working Conference on Eosinophil Disorders and Syndromes (Vienna, September 24–26, 2021) to discuss novel developments in the field encompassing all HE conditions, extending beyond hematopoietic neoplasms, and to refine criteria, definitions, and the classification of these disorders. Experts from the fields of dermatology, pathology, immunology, hematology, and laboratory medicine contributed to this project. All faculty members actively participated in pre‐conference and post‐conference discussions (March 2021 to March 2022). The outcomes of these discussions were formulated into consensus statements, which are summarized in this article. All faculty members contributed equally to discussions and manuscript preparation. The consensus‐reaching process is described in the Appendix S1.

## 2 EOSINOPHIL BIOLOGY AND NORMAL LABORATORY VALUES

Differentiation of normal eosinophils from their myelopoietic stem and progenitor cells is tightly controlled by a network of transcription factors, growth factors, and other cytokines.^12^, ^13^, ^14^, ^26^, ^27^, ^28^ Hematopoietic stem and progenitor cells that give rise to eosinophils are detectable in the bone marrow (BM) and peripheral blood (PB).^27^, ^29^, ^30^ In healthy adults, the major active pool of eosinophil progenitors resides in the BM. Mature eosinophils are also detected in normal BM aspirates, ranging between <1% and 6% in differential counts. The normal AEC in the PB ranges between 0.05 and 0.5 × 10^9^/L. Eosinophils are also found in the healthy thymus, spleen, lymph nodes, uterus, and the entire gastrointestinal tract distal to the esophagus. However, the physiological counts of eosinophils in these organs vary.

In common with other leukocytes, eosinophils derive from uncommitted CD34^+^ hematopoietic stem and progenitor cells.^26^, ^27^, ^29^ The most potent growth factors for eosinophils are IL‐5, granulocyte‐macrophage colony‐stimulating factor (GM‐CSF), and IL‐3.^6^, ^26^, ^27^, ^28^, ^29^, ^30^ These eosinopoietic cytokines are primarily produced by activated T cells, mast cells, type 2 innate lymphoid cells, and stromal cells, and trigger growth and survival as well as activation, adhesion, and migration of normal, reactive, and neoplastic eosinophils.^1^, ^2^, ^3^, ^12^, ^13^, ^14^, ^26^, ^27^, ^28^ Apart from the classical growth regulators mentioned above, several other cytokines and chemokines, such as transforming growth factors (TGF), platelet‐derived growth factors (PDGF), and CC/CXC ligands, can modulate eosinophil functions.^31^, ^32^, ^33^, ^34^ Eosinophil‐targeting cytokines and chemokines are summarized in Table S1. While reactive eosinophilia is induced by eosinopoietic cytokines, such as IL‐5, IL‐3, or GM‐CSF, clonal eosinophilia is typically triggered by rearrangements in certain oncogenic target genes, including *PDGFRA*, *PDGFRB*, fibroblast growth factor receptor‐1 (*FGFR1*), *JAK2, ABL1*, and *ETV6*.^6^, ^7^, ^8^, ^9^, ^10^, ^11^, ^14^, ^15^, ^16^, ^17^ The signaling networks downstream of ligand‐activated or/and oncogenic growth factor receptors in eosinophils (normal or neoplastic) are shown in Figure S1.

Eosinophils produce and store many biologically active molecules in their granules, including eosinophil peroxidase (EPX), eosinophil cationic protein (ECP), eosinophil major basic protein 1 (eMBP1), major basic protein 2, and numerous cytokines, including TGF‐ß (Table S2).^12^, ^13^, ^14^, ^35^, ^36^, ^37^, ^38^, ^39^, ^40^, ^41^, ^42^ In the setting of massive and persistent eosinophil activation, eosinophil‐derived (toxic) substances can cause substantial changes in the local microenvironment, resulting in organ damage, often in association with local inflammation, cytotoxicity, thromboembolic complications, and/or fibrosis.^1^, ^2^, ^3^, ^12^, ^13^, ^14^, ^42^, ^43^, ^44^ In patients with tissue HE and persistent eosinophil activation, marked deposition of eosinophil granule proteins, including eMBP1 and EPX, is usually found although staining for these eosinophil‐derived proteins is not standardized or available in most centers. Recommended routine stains for visualization and enumeration of eosinophils in organ specimens are the H&E, May–Grünwald–Giemsa, and Wright–Giemsa stains.^15^ Electron microscopy and immunostaining with antibodies to eosinophil granule proteins can provide information about deposition of eosinophil granules and their proteins when eosinophils are not identifiable as intact cells in tissue sections.^1^, ^15^, ^44^

## 3 UPDATED DEFINITION OF HYPEREOSINOPHILIA (HE)

Peripheral blood eosinophilia can occur as absolute blood eosinophilia (>0.5 × 10^9^/L), relative blood eosinophilia (>6% in differential counts) or combined absolute and relative eosinophilia (absolute >0.5 × 10^9^/L and >6%). Absolute PB eosinophilia can be divided into mild eosinophilia (0.5–1.49 × 10^9^/L), moderate hypereosinophilia (1.5–5.0 × 10^9^/L), and severe hypereosinophilia (>5.0 × 10^9^/L).^6^, ^7^, ^8^, ^9^, ^11^, ^15^, ^23^ As mentioned before, eosinophilia may be transient, episodic, or persistent.

Our faculty also discussed the issue that in some myeloid leukemias with extreme blood leukocytosis, eosinophils can be ≥1.5 × 10^9^/L blood but represent only a minority of leukocytes (<3%) and play no obvious pathogenic role. This holds true particularly for Ph‐chromosome‐positive CML and some AML variants. Therefore, our faculty concluded that the term HE should apply in CML and AML only when absolute and relative blood eosinophilia are present. Our proposed revised definition of HE for such leukemias is: persistent AEC ≥1.5 × 10^9^/L blood and ≥10% eosinophils in PB differential counts (Table 1).

**TABLE 1:** Definition of Hypereosinophilia (HE) and of the Hypereosinophilic Syndrome (HES)

Name/term	Abbreviation	Definition and criteria
Hypereosinophilia	HE	≥1.5 eosinophils ×10^9^/L peripheral blood on two examinations (interval ≥2 weeks).^a^
		Tissue HE may or may not be detected.
Tissue hypereosinophilia	Tissue HE	One or more of the following applies:
		a) the percentage of eosinophils in bone marrow section exceeds 20% of all nucleated cells, and/or
		b) a pathologist is of the opinion that tissue infiltration by eosinophils is extensive and/or
		c) marked deposition of eosinophil granule proteins is found (in the absence or presence of tissue infiltration by eosinophils)
Hypereosinophilic syndrome	HES	a) criteria for blood HE fulfilled and:
		b) organ damage and/or dysfunction attributable to tissue HE^b^ and:
		c) exclusion of other disorders or condition as major reason for organ damage
Tissue‐restricted HES^c^ (organ‐restricted HES)		a) tissue HE but criteria for blood HE not fulfilled and:
		b) organ damage and/or dysfunction attributable to tissue HE^b^ and:
		c) exclusion of other disorders or conditions as major reason for organ damage

Abbreviations: eMBP1, eosinophil major basic protein 1; EPX, eosinophil peroxidase; HE, hypereosinophilia; HES, hypereosinophilic syndrome(s).

^a^ In patients with chronic myeloid leukemia (CML) or acute myeloid leukemia (AML), HE is defined by an absolute eosinophil count of ≥1.5 × 10^9^/L peripheral blood and a relative eosinophil count of at least 10% (both for at least 2 weeks).

^b^ HE‐related organ damage (damage attributable to HE): organ dysfunction with marked tissue eosinophil infiltrates or/and extensive deposition of eosinophil‐derived proteins such as eMBP1 or EPX (in the presence or absence of marked tissue eosinophils) and typical clinical, histopathological and laboratory‐based signs of HE‐induced organ damage. When considering (establishing) the diagnosis HES is important to exclude all other etiologies as primary reason of organ damage.

^c^ When blood HE is not recorded in a patient with tissue HE and clear signs of HES, the (provisional) diagnosis of tissue‐restricted (organ‐restricted) HES can be established.

In our original definition of HE, “persistent” was defined as AEC >1.5 × 10^9^/L for at least 4 weeks.^15^ The WHO recently also proposed a 4‐week interval.^11^ In the Year 2021 Working Conference, this interval was again discussed. Based on our better understanding of the potential for rapidly deleterious clinical implications of certain driver genes and the availability of better (more rapid) diagnostic tests, our faculty agreed that the term “persistent” should apply to HE recorded on at least 2 occasions with a minimum “time‐interval” of 2 weeks.

Our faculty also discussed whether tissue HE should be defined with formal criteria and used to diagnose HE‐related pathologies and syndromes, including HES. After thorough discussion, our faculty concluded that the previously formulated criteria for tissue HE should be maintained and that the term “tissue HE” should be used in diagnostic reports documenting HE‐related pathology and syndromes.^15^, ^23^

Per the original definition, tissue HE is present when >1 of the following features is documented: (i) the percentage of eosinophils exceeds 20% of all nucleated cells in BM sections, (ii) a pathologist is of the opinion that tissue infiltration by eosinophils is extensive (massive) compared with “normal physiologic ranges,” or (iii) immunostaining reveals extensive extracellular deposition of eosinophil granule proteins, such as eMBP1 or EPX (Table 1).^15^ Although immunostaining for eosinophil granule proteins is not routinely available, when observed, it qualifies as a criterion for tissue HE even in the absence of marked (intact) eosinophil infiltration.^1^, ^15^

Of note, tissue HE can be detected in the absence of PB HE (e.g., in eosinophilic esophagitis or nasal polyposis), although in most instances, mild blood eosinophilia is also present. It is also important to state that the original definition of HES required PB HE.^15^ In other words, the diagnosis of HES could only be established when both PB HE and HE‐related organ damage were documented, irrespective of tissue HE.^15^ However, there are cases with tissue HE and associated organ damage resembling HES in which peripheral HE is absent. This occurs most commonly when only a single organ is involved. These patients should be labeled as “tissue‐restricted HES” or “organ‐restricted (mono‐organ) HES,” and most should be managed in the same way as those who have classically‐defined HES.

## 4 REFINED CRITERIA AND CLASSIFICATION OF HE VARIANTS

**FIGURE 1:**
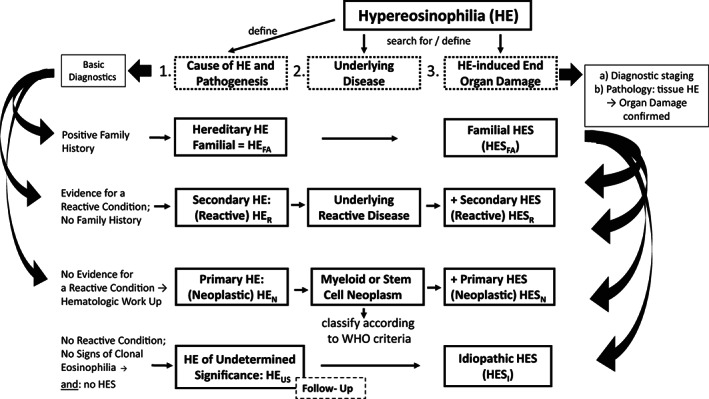
Diagnostic algorithm for patients with documented hypereosinophilia (HE). Patients with documented HE are examined for the presence of an underlying disease (etiology) and for the presence of eosinophil‐induced organ damage by applying basic diagnostics and specific staging investigations as well as specific molecular, laboratory, immunologic, hematologic, morphologic, and histopathologic investigations. The initial basic investigation includes a family history which may reveal familial HE (HE_FA_). In a next step, clinical and laboratory features of a reactive process are documented or excluded. In the case of a secondary reactive HE (HE_R_), the underlying disease process (inflammation, infection, tumor, others) needs to be defined. When no underlying reactive condition, no sign of clonality (neoplastic condition), and no signs of overt organ damage are found the provisional diagnosis is HE of unknown significance (HE_US_). These patients must be carefully monitored over time. When neoplastic HE (HE_N_) is detected, the final diagnosis of an underlying hematologic neoplasm must be determined by using WHO criteria and criteria provided by the ICOG‐EO group. When HE is accompanied by specific (HE‐induced) (multi)organ damage, the diagnosis of HES can be established. HES can occur in any type of HE and can present as secondary/reactive HES (HES_R_), primary/neoplastic HES (HES_N_), or idiopathic HES (HES_I_).

Based on clinical features and underlying etiology, HE can be divided into the following categories: familial (hereditary) HE (HE_FA_), HE of unknown significance (HE_US_), secondary (reactive) HE (HE_R_) where eosinophilia is non‐clonal and driven by overproduced cytokines, and primary (clonal, neoplastic) HE where the pathology is driven by neoplastic (clonal) eosinophils (HE_N_) (Table 2, Figure 1).^15^ It is important to note that these HE categories are not final diagnoses, but should prompt the physician to establish the etiology and pathology of the HE, and identify the underlying disorder. For example, a patient with HE_N_ may suffer from AML, *PDGFRA*‐rearranged MPN‐eo, or chronic eosinophilic leukemia (CEL).

**TABLE 2:** Classification of hypereosinophilia (HE)

Variant of HE	Abbreviation	Features
Hereditary (familial) HE	HE_FA_	Familial clustering, often evidence of a hereditary immunodeficiency (inborn errors of immunity with eosinophilia), no evidence of a reactive or neoplastic underlying disease, and no signs or symptoms indicative of HES
HE of unknown significance	HE_US_	No known underlying etiology of HE, no positive family history, no evidence of a reactive or neoplastic condition or disorder underlying HE, and no signs or symptoms indicative of HES
Secondary (reactive) HE	HE_R_	Underlying reactive condition or disease that explains HE, no evidence for a clonal bone marrow disease that explains HE^a^; and no signs or symptoms indicative of HES
Clonal (neoplastic) HE	HE_N_	Underlying stem cell, myeloid, or eosinophil neoplasm inducing HE^a^; no signs/symptoms indicative of HES

Abbreviations: HE, hypereosinophilia; HES, hypereosinophilic syndrome(s).

^a^ In clonal/neoplastic HE (HE_N_), eosinophils are considered to be clonal cells derived from neoplastic stem cells, whereas in reactive HE (HE_R_), eosinophils are considered to be reactive (non‐clonal) cells triggered by eosinopoietic cytokines such as interleukin‐5.

HE_R_ is by far the most common variant of HE. This category includes HE associated with infections (e.g., helminth infections), other reactive (inflammatory) diseases, lymphoid neoplasms (often T cell neoplasms producing eosinopoietic cytokines), and adverse drug reactions.

In all variants of HE, a detailed assessment of end organ function (by detailed imaging and other staging investigations as well as laboratory studies) is essential to rule out or to detect eosinophil‐related organ damage, which, if present, leads to a final diagnosis of HES.^15^ In the following paragraphs, we provide refined, updated criteria for the designated variants of HE. A diagnostic algorithm for patients with HE is depicted in Figure 1.

## 5 FAMILIAL HE (HE_FA_ ) = HEREDITARY HE

A number of hereditary conditions and syndromes are associated with familial HE (HE_FA_).^15^, ^23^, ^45^, ^46^, ^47^, ^48^, ^49^, ^50^ Most of these disorders are detected in childhood, and some are associated with immunodeficiency.^46^, ^47^, ^48^, ^49^ Well‐defined hereditary syndromes associated with HE and eosinophilia‐induced organ complications include Omenn syndrome, Wiskott‐Aldrich syndrome, Netherton syndrome, and Hyper‐IgE syndrome. A detailed description of these disorders and syndromes is beyond the scope of this article and is the subject of several recent reviews.^46^, ^47^, ^48^, ^49^ Germline mutations in certain driver genes of myelopoiesis, such as *JAK1*, are rare and may also be associated with hereditary (familial) HE.^50^ These conditions often manifest in adulthood, and not all family members develop typical symptoms of HES.^50^ Familial genetic disorders associated with HE are summarized in Table S3. Although patients with inborn immunodeficiency syndromes may also present with HE, most of these patients present with mild eosinophilia and do not develop typical manifestations of HES. Rare cases of familial clustering of HE have been described in the absence of a known genetic defect and/or the absence of symptoms. Examples include mitochondrial myopathies and a rare autosomal dominant variant of asymptomatic HE characterized by dysregulation of IL‐5 expression and rare progression to HES.^45^

## 6 HE OF UNKNOWN SIGNIFICANCE (HE_US_ )

When no familial clustering, underlying pathology, related molecular (genetic) abnormalities, or HE‐related organ damage is found in a patient presenting with HE, the provisional diagnosis “HE of unknown significance” (HE_US_) should be considered (Table 2, Figure 1).^15^, ^16^, ^23^, ^51^, ^52^ A diagnosis of HE_US_ requires (a) exclusion of an underlying disease or condition that can induce HE and (b) exclusion of HES. Close follow‐up of patients with HE_US_ is essential, since eosinophil‐related organ damage may develop after several months to years, and/or an underlying disease may become apparent over time with additional diagnostic testing. Should either of these occur, the diagnosis will change to another form of HE or HES (Figure 1).^15^, ^23^ Finally, mild eosinophilia (0.5–1.5 × 10^9^/L) not meeting criteria of HE may be associated with organ dysfunction or organ damage, and should, therefore, prompt the physician to initiate in‐depth investigations.^15^, ^16^, ^23^, ^51^, ^52^

## 7 REACTIVE HE (HE_R_ )

In HE_R_, eosinophils are presumed to be non‐clonal cells as demonstrated by exclusion of the presence of HE‐triggering driver mutations and related myeloid neoplasms. In most patients with HE_R_, eosinopoietic cytokines are considered to play a role in secondary eosinophil expansion and activation, and in many cases, overproduction of IL3, IL‐5, and/or GM‐CSF has been documented.^2^, ^3^, ^4^, ^5^, ^6^, ^12^, ^14^, ^53^ Whereas the underlying disease process can usually be identified and treated, the differential diagnosis is broad and includes inflammatory states, infections, autoimmune processes, and neoplastic disorders, such as solid tumors or lymphomas (Table S4).^2^, ^3^, ^4^, ^5^, ^6^, ^13^, ^14^, ^26^ In patients with suspected HE_R_ in whom no underlying reactive disease can be identified, exclusion of a myeloid or stem cell neoplasm (causing eosinophilia) is essential (see below).^15^, ^16^, ^23^ Sometimes, HE is followed for months or even years before a hematopoietic neoplasm is diagnosed.

In a subset of patients with HE_R_, one or more T cell subsets with an aberrant immunophenotype by flow cytometry (most commonly CD3^─^/CD4^+^) and increased production of type 2 cytokines, with or without evidence of a clonal T cell receptor (TCR) gene rearrangement, are found.^53^, ^54^, ^55^, ^56^, ^57^, ^58^ When signs of organ damage are also present, the lymphoid variant of HES (L‐HES), a special form of reactive HES, should be diagnosed.^15^, ^53^, ^54^, ^55^, ^56^, ^57^, ^58^ Importantly, detection of an isolated clonal TCR rearrangement in the absence of an abnormal T cell phenotype is not sufficient for diagnosis of L‐HES even if clinical criteria of HES are fulfilled.^15^, ^54^, ^55^ Patients with L‐HES are at increased risk for the development of a lymphoproliferative disease and should be followed accordingly.^58^, ^59^, ^60^

In a subset of (mostly pediatric) patients with B cell acute lymphoblastic leukemia, a specific translocation, t(5;14)(q31;q32), leads to juxtaposition of the *IgH* enhancer and the *IL‐3* gene, resulting in (over)production of IL‐3 by leukemic cells; these patients may also present with (reactive) HE.

## 8 NEOPLASTIC HE (HE_N_ ) = CLONAL HE

The World Health Organization (WHO) classification provides the basis for the delineation of hematopoietic neoplasms accompanied by clonal HE (Table S5).^8^, ^9^, ^10^, ^11^ In the 2016 and 2022‐updated WHO classifications, stem cell and myeloid neoplasms accompanied by eosinophilia are initially classified based on the presence of certain molecular markers, such as rearranged *PDGFRA* or *PDGFRB* (Table S5).^8^, ^9^, ^10^, ^11^ In many cases, a specific abnormality, such as *FIP1L1::PDGFRA*, is detected.^8^, ^9^, ^10^, ^11^, ^15^, ^16^, ^17^, ^18^, ^19^, ^20^, ^21^ In other patients, mutations in *JAK1* or *JAK2*, or other key signal‐transduction molecules, such as *STAT5*, are found.^61^, ^62^, ^63^ The 2022‐updated WHO classification and a recently proposed International Consensus Classification (ICC)^64^ include additional kinase fusion genes associated with HE in a newly named diagnostic category: “myeloid/lymphoid neoplasms with eosinophilia and tyrosine kinase gene fusions (MLN‐TK)” (Table S5).^11^

Standard evaluations to screen for such fusion genes include conventional cytogenetics, fluorescence in situ hybridization (FISH) and PCR. These assays are now also complemented by NGS‐based sequencing techniques to screen for the presence of additional mutations and rearrangements. Collectively, the techniques applied should cover the most common abnormalities involving *PDGFRA, PDGFRB, FGFR1, ETV6*, and *JAK2, BCR::ABL1*, AML‐specific fusion genes and *FLT3* rearrangements, as well as *JAK2* V617F and *KIT* D816V. Whole transcriptome sequencing and RNAseq are emerging technologies with broader scope, but are not yet in standard use as tests for myeloid neoplasms. In some hematopoietic neoplasms, such as B or T cell lymphomas, and plasma cell disorders, HE is usually reactive, whereas HE_R_ in myeloid neoplasms is very rare. An overview of mutations and fusion genes recurrently detected in patients with HE is provided in Table S5 and Figure S1.

**FIGURE 2:**
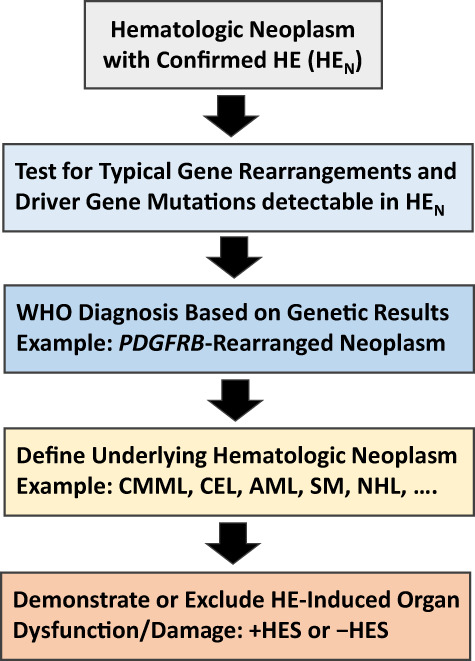
Diagnostic algorithm for patients suffering from hematologic diseases accompanied by clonal/neoplastic hypereosinophilia (HE_N_). In a first step, the presence of HE must be confirmed by measuring blood counts and the percentage of eosinophils by microscopy. In a next step, leukocytes are examined by PCR and next‐generation sequencing for the expression of certain gene variants known to be present in myeloid and stem cell neoplasms associated with HE. In addition, leukocytes from the bone marrow or blood are examined for specific abnormalities by conventional karyotyping and fluorescence in situ hybridization (FISH). At the same time, the underlying stem cell or myeloid neoplasm is defined by detailed studies of bone marrow and blood cells, including histomorphological, immunohistochemical, immunological, and biochemical analyses. When the patient is suffering from a lymphoid neoplasm (NHL), HE is considered to be non‐clonal and the diagnosis usually changes to HE_R_. In a final step, the patient is examined for the presence of signs and symptoms of specific organ involvement that could qualify as HE syndrome (HES). Here, it is of utmost importance to explore the case history and to ask the patient about previous potential HES‐related events, such as a thromboembolic complication. Abbreviations: +HES, with concomitant hypereosinophilic syndrome; AML, acute myeloid leukemia; CEL, chronic eosinophilic leukemia; CMML, chronic myelomonocytic leukemia; FISH, fluorescence in situ hybridization; HE, hypereosinophilia; −HES, without HES; NHL, Non Hodgkin lymphoma; PDGFR, platelet‐derived growth factor receptor; SM, systemic mastocytosis

In patients with HE_N_, the underlying neoplasm is defined based on morphologic, immunologic, and histomorphologic criteria provided by the WHO and ICOG‐EO.^6^, ^7^, ^8^, ^9^, ^10^, ^11^, ^15^, ^23^ These diagnoses range from myelodysplastic syndromes (MDS), MPN, and MDS/MPN overlap disorders to acute and chronic leukemias, and from systemic mastocytosis (SM) to various lymphoproliferative neoplasms (Table S5).^6^, ^7^, ^8^, ^9^, ^10^, ^11^ Once the molecular WHO entity and underlying neoplasm are defined, the presence or absence of an associated HES is determined in a final step (Figure 2).^15^, ^23^

Whereas this stepwise approach provides a logical framework for the diagnosis and classification of HE_N_, it is critical to define the molecular complexity of the underlying disease and the molecular targets as early as possible and to base the final diagnosis on histopathological and clinical parameters as well as the molecular features.^15^ This is important for several reasons. First, in many patients, multiple molecular defects are detectable even in the same founder‐clone (subclone‐formation), and it may be difficult to define the clinical impact of each individual molecular abnormality. Second, most molecular markers have been described in a wide range of neoplasms with differing pathologies, divergent clinical courses, and different responses to therapy.

Another important point is that the minimal allele burden (variant allele frequency, VAF) required to define some neoplasms is not well delineated, and sometimes, the role of a lesion expressed at low VAF (for example *JAK2* V617F at <2%) remains unclear in the setting of multiple concomitant mutations detected in the same patient. The WHO also regards a VAF below 2% as sub‐diagnostic in the context of eosinophil neoplasms such as CEL. Therefore, our faculty is of the opinion that the VAF (of each lesion) must be included in the final report and that the term clonal HE (or clonal HES) should be based on a minimal VAF of 3% (>2%). In cases where no HE‐related gene abnormality (fusion gene variant) is identified, patients can only be classified according to histopathological, morphological, immunological, and clinical parameters. Finally, the number of somatic mutations detectable in patients with myeloid (and other hematopoietic) neoplasms is increasing. Some of these mutations, such as age‐related mutations, are also detectable in healthy controls (often with a VAF <3%) and their detection in a patient with a myeloid neoplasm may play no dominant role in pathogenesis even if the VAF is rather high.

Based on these considerations, our faculty is of the opinion that the WHO and ICC classification of hematopoietic (stem cell and myeloid) neoplasms accompanied by HE should be followed,^11^, ^64^ but that the additional classification principles provided by the ICOG‐EO should also be applied.^15^, ^23^ Specifically, after the WHO‐related genetic markers are examined and the HE‐related primary pathology is documented (Figure 2), the presence or absence of HES should be addressed and the final diagnosis established according to a combination of WHO (ICC) and ICOG‐EO criteria (Figure 2).^15^, ^23^ Robust histopathological and morphological criteria should be applied to each individual case and used as the basis for the final hematological diagnosis. An illustrative example is eosinophilic leukemia associated with mastocytosis.^15^, ^23^

Based on the classification proposed by the ICOG‐EO, eosinophilic leukemias can be divided into chronic eosinophilic leukemia (CEL) and acute eosinophilic leukemia (AEL) (Table S5).^15^, ^23^ The WHO and ICC classifications include CEL among the classical MPN, but do not include AEL.^11^, ^64^ Per the ICOG‐EO proposal, CEL is diagnosed when the percentage of (clonal) eosinophils in the PB and/or BM is ≥30% and the percentage of myeloblasts is <20%.^15^ Certain driver‐related myeloid‐, stem cell‐ and mast cell neoplasms (including CML and JAK2 V617F+ MPN) must be excluded as primary trigger of HE before a diagnosis of CEL can be established, unless co‐existence of such a neoplasm with CEL is demonstrated with certainty based on detailed histopathological and molecular studies (Table S5). In the WHO proposal, the presence of any recurrent gene drivers of myeloid neoplasms (e.g., *BCR::ABL1*) or MLN‐TK precludes a diagnosis of CEL.^11^ In the ICOG‐EO proposal, AEL is diagnosed when the percentage of neoplastic eosinophils in the PB and/or BM is ≥30%, and the percentage of myeloblasts is ≥20% (Table S5).^15^ When HE is present and the percentage of eosinophils is below 30% in a patient with a stem cell‐derived or myeloid neoplasm, the final diagnosis is the WHO diagnosis together with the appendix “‐eo” (Table S5). For example, in a patient with *JAK2* V617F‐negative “primary myelofibrosis (MF)” in whom HE is present and the percentage of eosinophils is 18% (and thus below 30%), the final diagnosis is MPN‐eo (MF‐eo) (Table S5). However, if the percentage of eosinophils increases to 75% (and blast cells remain below 20%), the ICOG‐EO diagnosis would change to (secondary/post‐MF) CEL. Of note, the presence of eosinophilia is associated with poor prognosis (decreased survival) in several (chronic) myeloid neoplasms, including MDS and systemic mastocytosis (SM).^65^, ^66^, ^67^

The clinical impact of AEL remains uncertain given the rarity of this disease and the fact that the blast cell compartment (AML) is clinically more relevant than the impact of HE or HE‐induced organ dysfunction in patients with AEL. As mentioned, the current WHO and ICC classifications do not include AEL.^11^, ^64^

## 9 DEFINITION, CRITERIA, AND CLASSIFICATION OF HES

Based on the definition provided by the ICOG‐EO, HES is defined by (i) the presence of blood and/or tissue HE, (ii) HE‐associated organ damage, and (iii) exclusion of another underlying disorder or pathology as the primary driver of organ damage (Table 1).^15^, ^23^ The second (ii) and third (iii) criteria require detailed histopathological and clinical evaluation as well as imaging studies not only to document organ involvement but to determine that local infiltration of eosinophils and/or the toxic effects of eosinophil‐derived substances are the most likely cause.^15^, ^23^, ^68^, ^69^, ^70^ In patients with HES, clinically relevant organ damage can include one or more of the following features: (a) fibrosis (e.g., in the lungs, heart, digestive tract, and other organs), (b) thrombosis (thromboembolism) in various organ systems, (c) cutaneous (skin or mucosal) erythema, edema/angioedema, blisters, ulceration, or eczema, (d) pulmonary manifestations, (e) gastrointestinal involvement, (f) peripheral or central neuropathy with chronic or recurrent neurological deficit(s), (g) manifestations of eosinophilic vasculitis, and (h) other less common organ manifestations of HES (liver, pancreas, kidney, others).^15^, ^23^ Typical clinical features of HE‐related organ damage and thus HES are shown in Table S6. Whereas clinical and imaging studies (including radiological studies) are important and may often be diagnostic, in other cases only the pathologist will be able to confirm organ involvement by demonstrating the presence of tissue HE.^1^, ^2^, ^3^, ^15^, ^23^ In some instances, it may be difficult to establish a definite (causative) relationship between HE and the observed clinical manifestations either for technical reasons (e.g., the risks of obtaining an endomyocardial biopsy in an acutely ill patient or the need for urgent therapy), or because currently available investigations are not able to detect anomalies (e.g., central nervous system dysfunction may occur in the absence of overt abnormalities in imaging studies). Moreover, patients may experience non‐specific constitutional symptoms such as recurrent fever, malaise, fatigue, or myalgia, which may be severe but cannot be definitively related to eosinophil‐induced organ damage. In some cases, eosinophil involvement can reasonably be inferred from indirect but highly suggestive findings, such as the presence of classic findings of endomyocardial fibrosis on cardiac magnetic resonance imaging (Table S6). Furthermore, substantial improvement or regression of the symptomatology with treatment may provide indirect evidence that organ manifestations were triggered by HE.

All things considered, the term HES should be used for any patient in whom HE is clearly implicated in disease pathogenesis (organ damage), regardless of whether the HE results from a reactive process, a neoplastic process, or another underlying disease.^15^, ^23^ HES may be diagnosed at first presentation or during follow‐up. In particular, when specific organ damage is detected in a patient with HE, the diagnosis changes from HE to HES (Figure 1).^15^, ^23^ Moreover, eosinophil‐related organ damage in a single organ system may be sufficient to call the condition HES.^15^, ^23^ In such cases where the peripheral AEC is below the threshold defining blood HE, we propose the terms “tissue‐restricted HES,” or “organ‐restricted HES” (mono‐organ HES), even though the relative contribution of eosinophils to tissue damage may be difficult to ascertain, especially when other leukocyte subsets are also observed in histopathological analyses (e.g., increased mast cells and epithelial changes in eosinophilic esophagitis).

Patients with HE and HES are classified in a similar way based on underlying etiology with transition to the appropriate category of HES depending on the presence and nature of clinical manifestations.^15^ An updated classification of HES proposed by our ICOG‐EO group is shown in Table 3. The classification divides HES into familial/inherited HES (HES_FA_), idiopathic HES (HES_I_), reactive HES (HES_R_), and clonal/neoplastic HES (HES_N_) (Table 3). As mentioned before, the lymphoid variant of HES (L‐HES) is considered a reactive form of HES in which eosinophils are non‐clonal cells triggered by T cell‐derived cytokines.^54^, ^55^, ^56^, ^57^, ^58^, ^59^, ^60^ The diagnosis HES_FA_ is established when HE_FA_ is identified and the clinical criteria of HES (typical organ damage) are fulfilled.^15^, ^23^

**TABLE 3:** Classification of hypereosinophilic syndromes (HES) and related disorders/syndromes

Variant	Typical features
Familial HES (HES_FA_)	Familial clustering, very rare, IEI‐EO excluded^a^, typical end organ damage attributable to HE, no evidence of a reactive or neoplastic condition/disorder underlying HE
Idiopathic HES (HES_I_)	No underlying cause of HE, no evidence of a reactive or neoplastic condition/disorder underlying HE; and: end organ damage attributable to HE.
Primary (neoplastic) HES (HES_N_)	Underlying stem cell, myeloid, or eosinophil neoplasm classified according to WHO criteria^b^, and end organ damage attributable to HE. Eosinophils are neoplastic (clonal) cells; in many patients, rearranged/fusion variants of *PDGFRA*, *PDGFRB*, *FGFR1, JAK2, STAT5, FLT3, ABL1* or other driver genes, are found.
Secondary (reactive) HES (HES_R_)	Underlying condition/disease where eosinophils are considered non‐clonal cells, and HE is considered to be cytokine‐driven (HES_R_); and end organ damage attributable to HE.
Special variants of HES_R_:^c^	
a. Lymphoid variant of HES (L‐HES)	Abnormal clonal T cells are often detected, and HES‐related organ damage is found
b. Defined syndromes	
Episodic angioedema and eosinophilia (Gleich Syndrome)	Abnormal clonal T cells are often detected, angioedema, increased polyclonal IgM.
Eosinophilic granulomatosis with polyangiitis (eGPA) = Churg–Strauss syndrome	Polyangiitis, necrotizing angiitis, asthma, lung infiltrates; in a subset of patients, ANCA are detected (ANCA+ form of eGPA)
Eosinophilia myalgia syndrome (EMS)	Myalgia, muscle weakness, cramping, skin rash, dyspnea, fatigue.
IgG4‐related disease (IgG4‐RD)	Elevated serum IgG4 levels, HE, and HES‐like organ damages are found in about 30% of cases

Abbreviations: ANCA, anti‐neutrophil cytoplasmic antibodies; FGFR, fibroblast growth factor receptor; HE, hypereosinophilia; HES; hypereosinophilic syndrome; IEI‐EO, inborn errors of immunity with eosinophilia; PDGFR, platelet‐derived growth factor receptor.

^a^ The clinical symptoms and germline variants detectable in various forms of IEI‐EO are depicted in Table S3. These conditions may also present with organ dysfunction or even organ damage, but the organ damage in these patients is generally not related to HE—therefore, these cases are not classified as HES.

^b^ A more detailed description of stem cell and myeloid neoplasms associated with HE or HES is shown in Tables S4 and S5. In these cases, clonality of eosinophils is often difficult to demonstrate or is not examined. However, if a myeloid or stem cell neoplasm known to present typically with clonal HE, for example a myeloid neoplasm with *PDGFR*‐ or *FGFR*‐ rearrangement, is detected, HE can be regarded as clonal.

^c^ These syndromes may occur without fulfilling the formal criteria of HES. However, in most cases, the observed organopathy will qualify as HE‐related organ damage and thus as HES.

An important point is that HES should be differentiated from clinical syndromes associated with HE but that do not meet criteria for HES, such as some of the inborn errors of immunity and organ‐restricted inflammatory conditions where HE is present but does not play a major role in organ damage or dysfunction, and typical symptoms of HES are uncommon (Tables S3, S7 and S8).

Finally, it is of the utmost importance to delineate between the clinical syndrome of HES (defined by a symptom complex) and the underlying histopathological diagnosis. In fact, HES is neither a final diagnosis nor a defined immunological or hematologic disease. Rather, the contributing etiology and, thus, the underlying disease must be identified if possible in all patients with HES, and when no underlying disease is identified, the final diagnosis is HES_I._ ^15^ It is noteworthy that some patients with HE_US_, HE_R_ or HE_N_ do not develop clinical features of HES (organ damage) despite persistent HE over many years.^51^, ^52^

## 10 SPECIFIC SYNDROMES AND ORGAN‐SPECIFIC PATHOLOGIES ACCOMPANIED BY HE: CAN SOME OR ALL OF THESE CONDITIONS BE CLASSIFIED AS HES?

**FIGURE 3:**
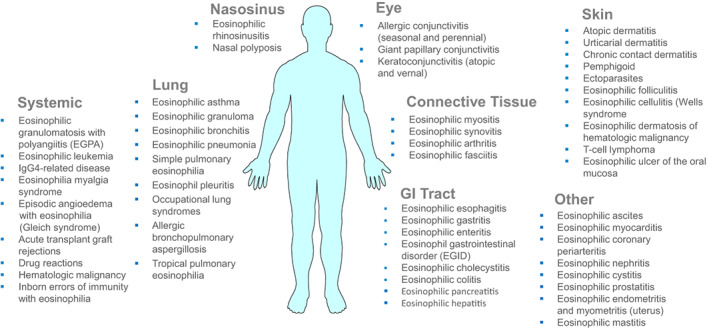
Organs potentially involved in patients with HE and HE‐related organ damage (HES). Compilation of disorders that are accompanied by eosinophilia and affect distinct organ systems. Organ involvement depends on the underlying etiology (disease), the exogenous (infectious), molecular and immunological triggers, and the number and degree of activation of infiltrating eosinophils. In patients with HES, multiple organs may be involved, and the same holds true for patients with defined syndromes, such as Gleich's syndrome or patients with eGPA. However, there are also patients with HES or other conditions accompanied by HE where only a single organ system is involved. Examples are eosinophilic colitis, eosinophilic gastritis, or eosinophilic pneumonia. In cases with primary (neoplastic) HES, the cardiovascular system is often affected, but cardiovascular complications may develop in any form of HES. Abbreviations: eGPA, eosinophilic granulomatosis with polyangiitis.

There are several specific syndromes and conditions associated with HE for which no underlying etiology or disease has been identified and/or the pathogenesis remains uncertain. These include patients with single organ‐restricted eosinophilic inflammation, such as eosinophilic colitis, eosinophilic gastritis, eosinophilic cystitis, eosinophilic hepatitis, several skin disorders, and certain forms of vasculitis (Figure 3, Tables S7 and S8). Our faculty is of the opinion that in many (or even most) of these conditions and syndromes, patients can be classified as HES_R_ provided HES criteria are fulfilled.

In another group of patients, a symptom complex or distinct molecular or immunological pattern can be detected. Examples include episodic angioedema with eosinophilia (Gleich Syndrome), eosinophilic granulomatosis with polyangiitis (eGPA), IgG4‐related disease (IgG4‐RD), and the eosinophilia‐myalgia syndrome (EMS) (Table 3, Table S8).^71^, ^72^ Gleich syndrome is characterized by angioedema, the presence of phenotypically aberrant clonal T cells, and increased polyclonal IgM.^72^ eGPA is a systemic eosinophilic vasculitis characterized by peripheral eosinophilia, asthma, and chronic rhinosinusitis with polyps. Anti‐neutrophil cytoplasmic antibodies (ANCA) are detected in approximately 40% of patients with eGPA. Given the similarity between the clinical manifestations of eGPA (and other HE‐related syndromes) and classical features of HES, our faculty is of the opinion that several of these conditions (syndromes) should be classified as HES provided that HES criteria are fulfilled. If this is not the case, then the final diagnosis is the named (known) syndrome and not HES.

## 11 CONCLUSIONS AND FUTURE PERSPECTIVES

Diagnosis and management of eosinophil‐associated disorders and related syndromes are an emerging challenge in the fields of clinical immunology, hematology, and pathology. With the recent availability of eosinophil‐depleting targeted drugs, it is now even more important to unify definitions and classifications for these conditions in an interdisciplinary effort. HE may develop in the context of various hematologic neoplasms and in certain reactive states. In all these patients, it is important to (i) document or exclude a related neoplastic or non‐neoplastic disease, and to (ii) document or exclude the presence of HE‐related organ damage (HES). Several immunological, serological, molecular, and cytogenetic markers are available to establish the nature of the underlying condition and, thus, help define the variant of HE and HES. In 2012, our consensus group proposed a comprehensive classification of eosinophil disorders together with diagnostic criteria. This proposal was based on a multidisciplinary approach involving the fields of allergy, immunology, hematology, pathology, and molecular medicine. Because of its multidisciplinary character and simple format, this concept has been widely accepted and is considered standard. However, recent developments in the field emerged and created a need to update and refine these concepts and diagnostic criteria. In the Year 2021 Working Conference on Eosinophil Disorders (Vienna, September 24–26, 2021), these developments were discussed and used to adjust diagnostic criteria, as well as definitions and classification of eosinophil disorders. As in 2011, these proposed criteria and definitions are based on a multidisciplinary approach and are in line with the previous ICOG‐EO consensus proposal and the 2016‐ and 2022‐updated classification of the WHO and ICC. We also define where the ICOG‐EO proposal complements or adds to the 2022‐updated WHO classification and ICC. Our updated definitions and criteria, along with our increasing knowledge about the etiology of HE should improve diagnosis, management, and prognosis of patients with eosinophil disorders in daily practice as well as in clinical trials.

## FUNDING INFORMATION

P.V. was supported by the Austrian Science Fund, grant F4704. B.S.B. was supported by National Institute of Allergy and Infectious Diseases grants U19 AI136443 and R21 AI159586. Research in the lab of H.U.S. is funded by the Swiss National Science Foundation, grant No. 310030_184816. FR was supported by Fonds de la Recherche Scientifique (FNRS) grant FC 54372. This work was supported in part by the Division of Intramural Research, NIAID, NIH (A.D.K.).

## CONFLICT OF INTEREST

The authors declare that they have no study‐related specific conflicts of interest. The authors declare the following conflicts of interest outside of this project: P.Valent received research grants from Pfizer, BMS/Celgene and AOP Orphan, and consultancy honoraria from Novartis, Pfizer, BMS/Celgene, Blueprint, Accord, and AOP Health. F.R. has received consultancy fees from Astra Zeneca and GlaxoSmithKline (GSK) and receives publication‐related royalty payments from UpToDate contributions. D.S. has been an investigator and/or advisor of AbbVie, AstraZeneca, Galderma, GSK, LEO Pharma, Elli Lilly, Novartis, Pfizer, Sanofi Genzyme. K.M.L. receives publication‐related royalty payments from UpToDate contributions, has a grant from Regeneron, receives royalties from Mayo Foundation, and holds patents for diagnosing, monitoring, and treating eosinophil‐related diseases. J.S. received consultancy honoraria from Novartis, GSK, Astra Zeneca and Blueprint. W.R.S. received consultancy honoraria from AbbVie, BMS/Celgene, Jazz, Novartis, Pfizer, StemLine, and Thermo Fisher. K.S. received consultancy honoraria from Novartis and Blueprint. P.Vandenberghe has received research support from Janssen Biotech and Pfizer via KU Leuven; and honoraria as speaker or advisory board member from Bristol‐Myers‐Squibb, Janssen Biotech, Miltenyi Biotec and Novartis Pharma. G.H. received honoraria from Novartis and Incyte. T.H. is part owner of the Munich Leukemia Laboratory (MLL). T.I.G has been a consultant for Incyte, Blueprint Medicines and Celgene/BMS. C.A. received consultancy honoraria from Novartis and Blueprint Medicines. B.S.B. receives publication‐related royalty payments from Elsevier and UpToDate®/Wolters Kluwer. He receives remuneration for consulting services (Third Harmonic Bio, Acelyrin Inc. and Lupagen) and for serving on the scientific advisory board of Allakos Inc., which he co‐founded, and owns stock in Allakos. He is a co‐inventor on existing Siglec‐8–related patents and thus receives royalty payments from Johns Hopkins University during development and potential sales of such products. The terms of this arrangement are being managed by Johns Hopkins University and Northwestern University in accordance with their conflict of interest policies. A.R. received research funding from Novartis and Blueprint and consultancy honoraria, speaker fees, and travel reimbursement from Novartis, Incyte, GSK, Blueprint, BMS/Celgene and Abbvie. J.G. received research funding from Incyte, Novartis, Kartos, Blueprint Medicines, Deciphera, Cogent Biosciences, Abbvie, Celgene, BMS, Protagonist Therapeutics, and advisory board/consulting honoraria from Incyte, Novartis, Kartos, Blueprint Medicines, Deciphera, Cogent Biosciences, Abbvie, Protagonist Therapeutics, and PharmaEssentia; he is also a PI and receives research funding for the FIGHT‐203 phase II study of pemigatinib in patients with *FGFR1*‐rearranged neoplasms; and is chair in the central review committee for the FIGHT‐203 study. M.A. received a research grant from Blueprint and honoraria from AB Science, Blueprint, and Novartis. H.U.S. is a consultant for GSK. G.J.G. has or had grants from National Institutes of Health, AstraZeneca, and GSK, is a science officer with NexEosBio, has a royalty sharing agreement with Teva, has received remuneration for consulting with AstraZeneca, GSK, Genentech, Knopp Biosciences, is on the Board of Directors for American Partnership for Eosinophilic Disorders (APFED), receives royalties from Mayo Foundation and holds patents for diagnosing, monitoring, and treating eosinophil‐related diseases. The authors declare no other conflict of interest.

## Supporting information


Appendix S1

Figure S1

## References

[1] Gleich GJ . Mechanisms of eosinophil‐associated inflammation. J Allergy Clin Immunol. 2000;105(4):651‐663. 10756213 10.1067/mai.2000.105712

[2] Simon D , Simon HU . Eosinophilic disorders. J Allergy Clin Immunol. 2007;119(6):1291‐1300. 17399779 10.1016/j.jaci.2007.02.010

[3] Akuthota P , Weller PF . Spectrum of eosinophilic end‐organ manifestations. Immunol Allergy Clin North Am. 2015;35(3):403‐411. 26209892 10.1016/j.iac.2015.04.002 PMC4515759

[4] Curtis C , Ogbogu P . Hypereosinophilic syndrome. Clin Rev Allergy Immunol. 2016;50(2):240‐251. 26475367 10.1007/s12016-015-8506-7

[5] Helbig G , Klion AD . Hypereosinophilic syndromes ‐ an enigmatic group of disorders with an intriguing clinical spectrum and challenging treatment. Blood Rev. 2021;49:100809. 33714638 10.1016/j.blre.2021.100809

[6] Valent P . Pathogenesis, classification, and therapy of eosinophilia and eosinophil disorders. Blood Rev. 2009;23(4):157‐165. 19246139 10.1016/j.blre.2009.01.001

[7] Bain BJ . Review: eosinophils and eosinophilic leukemia. Clin Adv Hematol Oncol. 2010;8(12):901‐903. 21326168

[8] Gotlib J . World Health Organization‐defined eosinophilic disorders: 2017 update on diagnosis, risk stratification, and management. Am J Hematol. 2017;92(11):1243‐1259. 29044676 10.1002/ajh.24880

[9] Reiter A , Gotlib J . Myeloid neoplasms with eosinophilia. Blood. 2017;129(6):704‐714. 28028030 10.1182/blood-2016-10-695973

[10] Shomali W , Gotlib J . World Health Organization‐defined eosinophilic disorders: 2022 update on diagnosis, risk stratification, and management. Am J Hematol. 2022;97(1):129‐148. 34533850 10.1002/ajh.26352

[11] Khoury JD , Solary E , Abla O , et al. The 5th edition of the World Health Organization classification of haematolymphoid tumours: myeloid and histiocytic/dendritic neoplasms. Leukemia. 2022;36(7):1703‐1719. 35732831 10.1038/s41375-022-01613-1 PMC9252913

[12] Weller PF , Spencer LA . Functions of tissue‐resident eosinophils. Nat Rev Immunol. 2017;17(12):746‐760. 28891557 10.1038/nri.2017.95 PMC5783317

[13] Klion AD , Ackerman SJ , Bochner BS . Contributions of eosinophils to human health and disease. Annu Rev Pathol. 2020;15:179‐209. 31977298 10.1146/annurev-pathmechdis-012419-032756 PMC7604902

[14] Valent P , Gleich GJ , Reiter A , et al. Pathogenesis and classification of eosinophil disorders: a review of recent developments in the field. Expert Rev Hematol. 2012;5(2):157‐176. 22475285 10.1586/ehm.11.81 PMC3625626

[15] Valent P , Klion A , Horny HP , et al. Contemporary consensus on criteria and classification of eosinophil disorders and related syndromes. J Allergy Clin Immunol. 2012;130(3):607‐612.e9. 22460074 10.1016/j.jaci.2012.02.019 PMC4091810

[16] Mattis DM , Wang SA , Lu CM . Contemporary classification and diagnostic evaluation of hypereosinophilia. Am J Clin Pathol. 2020;154(3):305‐318. 32525541 10.1093/ajcp/aqaa056

[17] Apperley JF , Gardembas M , Melo JV , et al. Response to imatinib mesylate in patients with chronic myeloproliferative diseases with rearrangements of the platelet‐derived growth factor receptor beta. N Engl J Med. 2002;347(7):481‐487. 12181402 10.1056/NEJMoa020150

[18] Cools J , DeAngelo DJ , Gotlib J , et al. A tyrosine kinase created by fusion of the PDGFRA and FIP1L1 genes as a therapeutic target of imatinib in idiopathic hypereosinophilic syndrome. N Engl J Med. 2003;348(13):1201‐1214. 12660384 10.1056/NEJMoa025217

[19] Jovanovic JV , Score J , Waghorn K , et al. Low‐dose imatinib mesylate leads to rapid induction of major molecular responses and achievement of complete molecular remission in FIP1L1‐PDGFRA‐positive chronic eosinophilic leukemia. Blood. 2007;109(11):4635‐4640. 17299092 10.1182/blood-2006-10-050054

[20] Metzgeroth G , Walz C , Erben P , et al. Safety and efficacy of imatinib in chronic eosinophilic leukaemia and hypereosinophilic syndrome: a phase‐II study. Br J Haematol. 2008;143(5):707‐715. 18950453 10.1111/j.1365-2141.2008.07294.x

[21] Rohmer J , Couteau‐Chardon A , Trichereau J , et al. Epidemiology, clinical picture and long‐term outcomes of FIP1L1‐PDGFRA‐positive myeloid neoplasm with eosinophilia: data from 151 patients. Am J Hematol. 2020;95(11):1314‐1323. 32720700 10.1002/ajh.25945

[22] Simon HU , Rothenberg ME , Bochner BS , et al. Refining the definition of hypereosinophilic syndrome. J Allergy Clin Immunol. 2010;126(1):45‐49. 20639008 10.1016/j.jaci.2010.03.042 PMC3400024

[23] Valent P , Klion AD , Rosenwasser LJ , et al. ICON: eosinophil disorders. World Allergy Organ J. 2012;5(12):174‐181. 23282419 10.1097/WOX.0b013e31827f4192 PMC3651188

[24] Klion AD . Eosinophilia: a pragmatic approach to diagnosis and treatment. Hematology Am Soc Hematol Educ Program. 2015;2015:92‐97. 26637706 10.1182/asheducation-2015.1.92

[25] Kahn JE , Groh M , Lefèvre G . (A critical appraisal of) classification of hypereosinophilic disorders. Front Med (Lausanne). 2017;4:216. 29259972 10.3389/fmed.2017.00216 PMC5723313

[26] Fulkerson PC , Rothenberg ME . Eosinophil development, disease involvement, and therapeutic suppression. Adv Immunol. 2018;138:1‐34. 29731004 10.1016/bs.ai.2018.03.001

[27] Salter BM , Ju X , Sehmi R . Eosinophil lineage‐committed progenitors as a therapeutic target for asthma. Cell. 2021;10(2):412. 10.3390/cells10020412 PMC7920418 33669458

[28] Renz H , Bachert C , Berek C , et al. Physiology and pathology of eosinophils: recent developments: summary of the focus workshop organized by DGAKI. Scand J Immunol. 2021;93(6):e13032. 33624312 10.1111/sji.13032 PMC11475402

[29] Leary AG , Ogawa M . Identification of pure and mixed basophil colonies in culture of human peripheral blood and marrow cells. Blood. 1984;64(1):78‐83. 6234038

[30] Sehmi R , Baatjes AJ , Denburg JA . Hemopoietic progenitor cells and hemopoietic factors: potential targets for treatment of allergic inflammatory diseases. Curr Drug Targets Inflamm Allergy. 2003;2(4):271‐278. 14561146 10.2174/1568010033484007

[31] Bach MK , Brashler JR , Stout BK , et al. Activation of human eosinophils by platelet‐derived growth factor. Int Arch Allergy Immunol. 1992;97(2):121‐129. 1316315 10.1159/000236107

[32] Noso N , Proost P , Van Damme J , Schröder JM . Human monocyte chemotactic proteins‐2 and 3 (MCP‐2 and MCP‐3) attract human eosinophils and desensitize the chemotactic responses towards RANTES. Biochem Biophys Res Commun. 1994;200(3):1470‐1476. 7514401 10.1006/bbrc.1994.1616

[33] Rothenberg ME , Ownbey R , Mehlhop PD , et al. Eotaxin triggers eosinophil‐selective chemotaxis and calcium flux via a distinct receptor and induces pulmonary eosinophilia in the presence of interleukin 5 in mice. Mol Med. 1996;2(3):334‐348. 8784786 PMC2230145

[34] Menzies‐Gow A , Ying S , Sabroe I , et al. Eotaxin (CCL11) and eotaxin‐2 (CCL24) induce recruitment of eosinophils, basophils, neutrophils, and macrophages as well as features of early‐ and late‐phase allergic reactions following cutaneous injection in human atopic and nonatopic volunteers. J Immunol. 2002;169(5):2712‐2718. 12193745 10.4049/jimmunol.169.5.2712

[35] Lehrer RI , Szklarek D , Barton A , Ganz T , Hamann KJ , Gleich GJ . Antibacterial properties of eosinophil major basic protein and eosinophil cationic protein. J Immunol. 1989;142(12):4428‐4434. 2656865

[36] Hamann KJ , Barker RL , Ten RM , Gleich GJ . The molecular biology of eosinophil granule proteins. Int Arch Allergy Appl Immunol. 1991;94(1‐4):202‐209. 1657792 10.1159/000235362

[37] Moqbel R , Levi‐Schaffer F , Kay AB . Cytokine generation by eosinophils. J Allergy Clin Immunol. 1994;94(6):1183‐1188. 7798558 10.1016/0091-6749(94)90330-1

[38] Kay AB , Barata L , Meng Q , Durham SR , Ying S . Eosinophils and eosinophil‐associated cytokines in allergic inflammation. Int Arch Allergy Immunol. 1997;113(1‐3):196‐199. 9130521 10.1159/000237545

[39] Spencer LA , Szela CT , Perez SA , et al. Human eosinophils constitutively express multiple Th1, Th2 and immunoregulatory cytokines that are secreted rapidly and differentially. J Leukoc Biol. 2009;85(1):117‐123. 18840671 10.1189/jlb.0108058 PMC2626765

[40] Davoine F , Lacy P . Eosinophil cytokines, chemokines, and growth factors: emerging roles in immunity. Front Immunol. 2014;5:570. 25426119 10.3389/fimmu.2014.00570 PMC4225839

[41] Sastre B , Rodrigo‐Muñoz JM , Garcia‐Sanchez DA , Cañas JA , Del Pozo V . Eosinophils: old players in a new game. J Investig Allergol Clin Immunol. 2018;28(5):289‐304. 10.18176/jiaci.0295 30059011

[42] Kanda A , Yasutaka Y , Van Bui D , et al. Multiple biological aspects of eosinophils in host defense, eosinophil‐associated diseases, immunoregulation, and homeostasis: is their role beneficial, detrimental, regulator, or bystander? Biol Pharm Bull. 2020;43(1):20‐30. 31902927 10.1248/bpb.b19-00892

[43] Jackson DJ , Akuthota P , Roufosse F . Eosinophils and eosinophilic immune dysfunction in health and disease. Eur Respir Rev. 2022;31(163):210150. 35082127 10.1183/16000617.0150-2021 PMC9489126

[44] Saffari H , Hoffman LH , Peterson KA , et al. Electron microscopy elucidates eosinophil degranulation patterns in patients with eosinophilic esophagitis. J Allergy Clin Immunol. 2014;133(6):1728‐1734.e1. 24439077 10.1016/j.jaci.2013.11.024

[45] Prakash Babu S , Chen YK , Bonne‐Annee S , et al. Dysregulation of interleukin 5 expression in familial eosinophilia. Allergy. 2017;72(9):1338‐1345. 28226398 10.1111/all.13146 PMC5546948

[46] Engelhardt KR , Gertz ME , Keles S , et al. The extended clinical phenotype of 64 patients with dedicator of cytokinesis 8 deficiency. J Allergy Clin Immunol. 2015;136(2):402‐412. 25724123 10.1016/j.jaci.2014.12.1945 PMC4530066

[47] Olbrich P , Ortiz Aljaro P , Freeman AF . Eosinophilia associated with immune deficiency. J Allergy Clin Immunol Pract. 2022;10(5):1140‐1153. 35227935 10.1016/j.jaip.2022.02.016

[48] Navabi B , Upton JE . Primary immunodeficiencies associated with eosinophilia. Allergy Asthma Clin Immunol. 2016;12:27. 27222657 10.1186/s13223-016-0130-4 PMC4878059

[49] Okamoto K , Morio T . Inborn errors of immunity with eosinophilia. Allergol Int. 2021;70(4):415‐420. 34456137 10.1016/j.alit.2021.08.008

[50] Del Bel KL , Ragotte RJ , Saferali A , et al. JAK1 gain‐of‐function causes an autosomal dominant immune dysregulatory and hypereosinophilic syndrome. J Allergy Clin Immunol. 2017;139(6):2016‐2020.e5. 28111307 10.1016/j.jaci.2016.12.957

[51] Helbig G , Kyrcz‐Krzemień S . Idiopathic, asymptomatic, and durable blood hypereosinophilia‐still many unknowns. J Allergy Clin Immunol. 2014;133(3):932‐933. 24439081 10.1016/j.jaci.2013.12.023

[52] Chen YY , Khoury P , Ware JM , et al. Marked and persistent eosinophilia in the absence of clinical manifestations. J Allergy Clin Immunol. 2014;133(4):1195‐1202. 23987798 10.1016/j.jaci.2013.06.037 PMC3935986

[53] Simon HU , Plötz SG , Dummer R , Blaser K . Abnormal clones of T cells producing interleukin‐5 in idiopathic eosinophilia. N Engl J Med. 1999;341(15):1112‐1120. 10511609 10.1056/NEJM199910073411503

[54] Roufosse F , Cogan E , Goldman M . Lymphocytic variant hypereosinophilic syndromes. Immunol Allergy Clin North Am. 2007;27(3):389‐413. 17868856 10.1016/j.iac.2007.07.002

[55] Roufosse F . Hypereosinophilic syndrome variants: diagnostic and therapeutic considerations. Haematologica. 2009;94(9):1188‐1193. 19734412 10.3324/haematol.2009.010421 PMC2738708

[56] Carpentier C , Schandené L , Dewispelaere L , Heimann P , Cogan E , Roufosse F . CD3‐CD4+ lymphocytic variant hypereosinophilic syndrome: diagnostic tools revisited. J Allergy Clin Immunol Pract. 2021;9(6):2426‐2439.e7. 33545400 10.1016/j.jaip.2021.01.030

[57] Ledoult E , Groh M , Kahn JE , et al. Assessment of T‐cell polarization on the basis of surface marker expression: diagnosis and potential therapeutic implications in lymphocytic variant hypereosinophilic syndrome. J Allergy Clin Immunol Pract. 2020;8(3):1110‐1114.e2. 31525539 10.1016/j.jaip.2019.08.049

[58] Lefèvre G , Copin MC , Roumier C , et al. CD3‐CD4+ lymphoid variant of hypereosinophilic syndrome: nodal and extranodal histopathological and immunophenotypic features of a peripheral indolent clonal T‐cell lymphoproliferative disorder. Haematologica. 2015;100(8):1086‐1095. 25682606 10.3324/haematol.2014.118042 PMC5004425

[59] Carpentier C , Verbanck S , Schandené L , et al. Eosinophilia associated with CD3‐CD4+ T cells: characterization and outcome of a single‐center cohort of 26 patients. Front Immunol. 2020;11:1765. 32849632 10.3389/fimmu.2020.01765 PMC7432433

[60] Shi Y , Wang C . What we have learned about lymphocytic variant hypereosinophilic syndrome: a systematic literature review. Clin Immunol. 2022;237:108982. 35307610 10.1016/j.clim.2022.108982

[61] Ma CA , Xi L , Cauff B , et al. Somatic STAT5b gain‐of‐function mutations in early onset nonclonal eosinophilia, urticaria, dermatitis, and diarrhea. Blood. 2017;129(5):650‐653. 27956386 10.1182/blood-2016-09-737817 PMC5290989

[62] Cross NCP , Hoade Y , Tapper WJ , et al. Recurrent activating STAT5B N642H mutation in myeloid neoplasms with eosinophilia. Leukemia. 2019;33(2):415‐425. 30573779 10.1038/s41375-018-0342-3 PMC6365490

[63] Shomali W , Damnernsawad A , Theparee T , et al. A novel activating *JAK1* mutation in chronic eosinophilic leukemia. Blood Adv. 2021;5(18):3581‐3586. 34496019 10.1182/bloodadvances.2021004237 PMC8945578

[64] Tzankov A , Reichard KK , Hasserjian RP , Arber DA , Orazi A , Wang SA . Updates on eosinophilic disorders. Virchows Arch. 2022; in press. 10.1007/s00428-022-03402-8 36068374

[65] Matsushima T , Handa H , Yokohama A , et al. Prevalence and clinical characteristics of myelodysplastic syndrome with bone marrow eosinophilia or basophilia. Blood. 2003;101(9):3386‐3390. 12506028 10.1182/blood-2002-03-0947

[66] Wimazal F , Germing U , Kundi M , et al. Evaluation of the prognostic significance of eosinophilia and basophilia in a larger cohort of patients with myelodysplastic syndromes. Cancer. 2010;116(10):2372‐2381. 20209617 10.1002/cncr.25036

[67] Kluin‐Nelemans HC , Reiter A , Illerhaus A , et al. Prognostic impact of eosinophils in mastocytosis: analysis of 2350 patients collected in the ECNM registry. Leukemia. 2020;34(4):1090‐1101. 31740811 10.1038/s41375-019-0632-4 PMC7115841

[68] Pineton de Chambrun G , Desreumaux P , Cortot A . Eosinophilic enteritis. Dig Dis. 2015;33(2):183‐189. 25925921 10.1159/000369540

[69] Brambatti M , Matassini MV , Adler ED , Klingel K , Camici PG , Ammirati E . Eosinophilic myocarditis: characteristics, treatment, and outcomes. J Am Coll Cardiol. 2017;70(19):2363‐2375. 29096807 10.1016/j.jacc.2017.09.023

[70] Tennenbaum J , Groh M , Venditti L , et al. FIP1L1‐PDGFRA‐associated hypereosinophilic syndrome as a treatable cause of watershed infarction. Stroke. 2021;52(10):e605‐e609. 34304603 10.1161/STROKEAHA.121.034191

[71] Moussiegt A , Müller R , Ebbo M , et al. IgG4‐related disease and hypereosinophilic syndrome: overlapping phenotypes. Autoimmun Rev. 2021;20(9):102889. 34237420 10.1016/j.autrev.2021.102889

[72] Khoury P , Herold J , Alpaugh A , et al. Episodic angioedema with eosinophilia (Gleich syndrome) is a multilineage cell cycling disorder. Haematologica. 2015;100:300‐307. 25527564 10.3324/haematol.2013.091264 PMC4349267

