# Supplementary material for: Proposed refined diagnostic criteria and classification of eosinophil disorders and related syndromes
Source: Allergy. 2022 Oct 19;78(1):47–59. doi: 10.1111/all.15544 (PMC9797433; doi:10.1111/all.15544)
Supplement: Supplementary file 1 — Appendix S1 [file ALL-78-47-s001.docx]

**Supplemental File to:**

**Proposed Refined Diagnostic Criteria and Classification of Eosinophil Disorders and Related Syndromes**

Peter Valent^1,2^*, Amy D. Klion^3^, Florence Roufosse^4^, Dagmar Simon^5^, Georgia Metzgeroth^6^, Kristin M. Leiferman^7^, Juliana Schwaab^6^, Joseph H. Butterfield^8^, Wolfgang R. Sperr^1,2^, Karl Sotlar^9^, Peter Vandenberghe^10^, Gregor Hoermann^11^, Torsten Haferlach^11^, Richard Moriggl^l2^, Tracy I. George^13^, Cem Akin^14^, Bruce S. Bochner^15^, Jason Gotlib^16^, Andreas Reiter^6^,

Hans-Peter Horny^17^, Michel Arock^18^, Hans-Uwe Simon^19,20^, Gerald J. Gleich^21^

^1^Department of Internal Medicine I, Division of Hematology & Hemostaseology, Medical University of Vienna, Austria; ^2^Ludwig Boltzmann Institute for Hematology and Oncology, Medical University of Vienna, Austria; ^3^Human Eosinophil Section, Laboratory of Parasitic Diseases, NIH/NIAID, Bethesda, MD, USA; ^4^Department of Internal Medicine Erasme Hospital, Université Libre de Bruxelles, Brussels, Belgium; ^5^Department of Dermatology, Inselspital Bern, University of Bern, Switzerland; ^6^Department of Hematology and Oncology, University Hospital Mannheim - Heidelberg University, Germany; ^7^Department of Dermatology, University of Utah Health Sciences Center, Salt Lake City, UT, USA; ^8^Division of Allergic Diseases, Mayo Clinic, Rochester, MN, USA; ^9^Institute of Pathology, University Hospital Salzburg, Paracelsus Medical University, Salzburg, Austria; ^10^Division of Hematology, University Hospital Leuven and Department of Human Genetics, KU Leuven, Belgium; ^11^MLL Munich Leukemia Laboratory, Munich, Germany; ^12^Institute of Animal Breeding and Genetics, University of Veterinary Medicine, Vienna, Austria; ^13^Department of Pathology, University of Utah, Salt Lake City, UT, USA; ^14^Division of Allergy and Clinical Immunology, University of Michigan, Ann Arbor, MI, USA; ^15^Northwestern University Feinberg School of Medicine, Division of Allergy and Immunology, Chicago, IL, USA; ^16^Stanford Cancer Institute/Stanford University School of Medicine, Stanford, CA, USA; ^17^Institute of Pathology, Ludwig Maximilian University Munich (LMU), Munich, Germany; ^18^Department of Hematological Biology, Pitié-Salpêtrière Hospital, Pierre et Marie Curie University (UPMC), Paris, France; ^19^Institute of Pharmacology, University of Bern, Bern, Switzerland; ^20^Institute of Biochemistry, Brandenburg Medical School, Neuruppin, Germany; ^21^Departments of Dermatology and Medicine, University of Utah Health Sciences Center,

Salt Lake City, UT, USA.

Short Title: Criteria and classification of eosinophil disorders

*Correspondence:

Peter Valent, M.D.

Department of Medicine I

Division of Hematology & Hemostaseology and

Ludwig Boltzmann Institute for Hematology and Oncology

Medical University of Vienna

Währinger Gürtel 18-20, A-1090 Vienna, Austria

Phone: 43 1 40400 4415; Fax: 43 1 40040 4030

E-mail: [peter.valent@meduniwien.ac.at](mailto:peter.valent@meduniwien.ac.at)

**Information on the Working Conference and the Consensus Discussion**

The Working Conference on Eosinophil Disorders and Related Syndromes (official title: Year 2021 Working Conference on Eosinophil Disorders and Related Syndromes) was organized in Vienna in September 2021 (September 24-26, 2021) by the Medical University of Vienna in collaboration with the Vienna Cancer Stem Cell Club and the Ludwig Boltzmann Institute for Hematology and Oncology (LBI HO). The project included an in-depth discussion on eosinophil disorders and related conditions and lasted from March 2021 until March 2022. The discussion phase was split into a pre-conference phase (via e-mails and smaller preparative meetings), the conference discussion at the Working Conference, and a post-conference discussion phase (September 2021 until March 2022). Because of the corona virus pandemic, the conference was organized in hybrid mode (on-site plus web-based). The consensus discussion and the consensus decision-making process were organized in accordance with published guidelines.^1^

In the final discussion round, the paper-draft was discussed and adjusted based on input provided by all faculty members and available information. Open discussion points were reviewed and discussed in the faculty (consensus group = co-authors) until an unanimous agreement was obtained or no consensus was reached. Only those statements, criteria, and definitions that are based on a 100% consensus among all faculty members were included in the final document.

The working definitions, criteria, and classification were based on our initial proposal (ICOG-EO and WAO) published in 2012^2,3^ and the proposal to classify hematologic neoplasms with eosinophilia proposed by the World Health Classification (WHO).^4^ Gene variants, including fusion genes, are provided according to the Human Genome (HUGO) Gene Nomenclature Committee (HGNC) recommendations published recently.^5^

The final document and its content were approved by all faculty members (all co-authors) before submission. All actively contributing faculty members are included as co-authors in the final document.

**References**

1. Graham R, Mancher M, Wolman DM, Greenfield S, Steinberg E, eds; Institute of Medicine; Board on Health Care Services; Committee on Standards for Developing Trustworthy Clinical Practice Guidelines. Clinical Practice Guidelines We Can Trust. Washington, DC: National Academies Press; 2011.

2. Valent P, Klion A, Horny HP, et al. Contemporary consensus on criteria and classification of eosinophil disorders and related syndromes. *J Allergy Clin Immunol*. 2012;130(3):607-612.e9.

3. Valent P, Klion AD, Rosenwasser LJ, et al. ICON: Eosinophil Disorders. *World Allergy Organ J*. 2012;5(12):174-181.

4. Gotlib J. World Health Organization-defined eosinophilic disorders: 2017 update on diagnosis, risk stratification, and management. *Am J Hematol*. 2017;92(11):1243-1259.

5. Bruford EA, Antonescu CR, Carroll AJ, et al. HUGO Gene Nomenclature Committee (HGNC) recommendations for the designation of gene fusions. *Leukemia*. 2021;35(11):3040-3043.

6. Navabi B, Upton JE. Primary immunodeficiencies associated with eosinophilia. *Allergy Asthma Clin Immunol*. 2016;12:27.

7. Okamoto K, Morio T. Inborn errors of immunity with eosinophilia. *Allergol Int*. 2021;70(4):415-420.

8. Leiferman KM, Peters MS. Eosinophil-related disease and the skin. *J Allergy Clin Immunol Pract*. 2018;6(5):1462-1482.e6.

**Supplemental Tables**

Supplemental Table S1

Eosinophil-Targeting Cytokines and Ligands, and Interacting Surface Receptors

-----------------------------------------------------------------------------------------------------------------

Ligands/ Effects on eosinophils and/or

cytokines eosinophil precursor cells Receptor (R)

-----------------------------------------------------------------------------------------------------------------

Classical growth factors:

IL-3 Differentiation, survival, adhesion, IL-3R

Migration, activation, priming (CD123+CD131)

IL-5 Differentiation, survival, adhesion, IL-5R

Migration, activation, priming (CD125+CD131)

GM-CSF Differentiation, survival, adhesion, GM-CSFR

Migration, activation, priming (CD116+CD131)

Other cytokines potentially promoting growth and survival:

PDGF Survival*, activation? PDGFRA/B

FGF Survival*, activation? FGFR1

Activation and/or migration-inducing cytokines:

IL-2 Activation, priming IL-2RA/CD25

IL-4 Priming for chemotaxins IL-4R/CD124

IL-13 Activation? IL-13R

IL-16 Activation, priming CD4, CD9(?), CCR3

IL-25 Activation IL-25R

IL-27 Activation IL-27R

IL-31 Chemotaxis, activation IL-31R

IL-33 Activation, adhesion, migration IL-33R/ST2

VEGF Chemotaxis, activation VEGFR-1/FLT-1

Angiopoietin-1 Chemotaxis, activation? Tie-2/TEK

Other activating and/or migration-inducing factors and chemokines:

C3a, C5a Chemotaxis, activation C3aR, C5aR

PGD_2_ Chemotaxis, activation CRTH2 (PGD_2_R)

PAF Chemotaxis, activation PAF-R

SDF-1 (CXCL12) Chemotaxis** CXCR4 (CD184)

RANTES (CCL5) Chemotaxis, activation CCR3

MCP-3 (CCL7) Chemotaxis, activation CCR3

MCP-4 (CCL13) Chemotaxis, activation CCR3

Eotaxin (CCL11) Chemotaxis, activation CCR3

Eotaxin-2 (CCL24) Chemotaxis, activation CCR3

Eotaxin-3 (CCL26) Chemotaxis, activation CCR3

Inhibitory cytokines:

TGFß1 Inhibitory (growth, activation) TGFß1R

TGFß2 Inhibitory (growth, activation) TGFß2R

IFN-alpha Inhibitory (growth) IFN-alpha-R

IFN-gamma Inhibitory (growth, migration) IFN-gamma-R

IL-10 Inhibitory (activation, survival) IL-10R

IL-12 Inhibitory (activation) IL-12R

Siglec-8L Inhibitory (survival) Siglec-8

-----------------------------------------------------------------------------------------------------------------

*In patients with myeloid or stem cell neoplasms accompanied by HE oncogenic mutant forms of *PDGFR* or *FGFR* are often detected in neoplastic precursor cells. In these cells, eosinophil differentiation is triggered primarily by such oncogenic mutant forms of PDGFR or FGFR. **SDF-1 is a strong chemotactic factor for normal and neoplastic eosinophils expressing CXCR4 (CD184). Abbreviations: IL, interleukin; GM-CSF, granulocyte/macrophage colony-stimulating factor; PDGF, platelet-derived growth factor; FGF, fibroblast growth factor; VEGF, vascular endothelial growth factor; C3a, complement factor 3a; C5a, complement factor 5a; PGD_2_, prostaglandin D2; PAF, platelet-activating factor; SDF-1, stroma derived factor-1; CCL, CC chemokine ligand; CCR, CC chemokine receptor; CXCL, CXC chemokine ligand; CXCR, CXC chemokine receptor; RANTES, regulated upon activation, normal T cell expressed and secreted; MCP, monocyte chemotactic protein; TGF, transforming growth factor; IFN, interferon; R, receptor.

Supplemental Table S2

Eosinophil Products and their Potential Impact in the Etiology of HE and HES

----------------------------------------------------------------------------------------------------------------------

Effects potentially relevant to

Eosinophil Product HE-related organ damage = HES*

----------------------------------------------------------------------------------------------------------------------

Cytokines/interleukins:

GM-CSF Leukocyte/eosinophil expansion, survival,

activation, migration, and accumulation

IL-1 Endothelial cell activation, inflammation

IL-2 Activation of T lymphocytes

IL-3 Eosinophil accumulation and activation

IL-4 B cell maturation and mast cell development,

endothelial VCAM-1 induction

IL-5 Eosinophil expansion, survival, migration,

accumulation and activation

IL-6 Lymphocyte maturation/activation

IL-8 Leukocyte recruitment/activation

IL-13 Bronchial hyper-reactivity, mucus production,

B cell maturation, endothelial VCAM-1 induction

IL-31 Pruritus, inflammation

TGF-alpha Fibrosis, growth inhibition

TGF-beta Fibrosis, growth inhibition

TNF-alpha Endothelial activation, inflammation, cachexia

OSM Fibrosis, angiogenesis**

SCF Mast cell accumulation/activation/survival

Chemokine ligands:

Eotaxin (CCL11) Eosinophil recruitment

MIP-1-alpha (CCL3) Leukocyte recruitment and activation

RANTES (CCL5) Leukocyte recruitment and activation

Neurotrophins:

Nerve growth factor (NGF) Enhanced neurologic responses

Neurotrophin-3 Enhanced neurologic responses

Eosinophil-derived basic proteins:

Eosinophil cationic protein (ECP) Direct toxic effects, mucus secretion, fibrosis

Eosinophil-derived neurotoxin (EDN) Direct toxic effects, TLR-2 ligand effects, RNase

Eosinophil peroxidase (EPX) Direct toxic effects, leukocyte activation

Eosinophil major basic protein 1 (eMBP1) Direct toxic effects, leukocyte activation,

thrombosis

Eosinophil major basic protein 2 (eMBP2) Direct toxic effects, leukocyte activation

Toxic and immunoregulatory enzymes:

Acid phosphatase Direct toxic effect

Arylsulphatase B Lysosomal hydrolase

Catalase Direct toxic effects

Hexosaminidase Direct toxic effects

Histaminase Histamine degradation

Lysophospholipase Direct toxic effects

Nonspecific esterases Direct toxic effects

Phospholipase D LFA-dependent adhesion

Membrane-derived lipid compounds:

LTC_4_ Mucus secretion

PAF Bronchoconstriction, edema formation

PGE_1_ & PGE_2_ Diverse effects on platelets, endothelial cells,

fibroblasts and other tissue cells

15–HETE Diverse effects on blood and tissue cells

TXB_2_ Platelet aggregation

Thrombosis-triggering factors:

HE-related DNA traps Direct toxic and pro-thrombotic effects

PAI-2 Anti-fibrinolytic and pro-thrombotic effects

------------------------------------------------------------------------------------------------------------------------

*Eosinophil-derived mediators, proteins, and enzymes and their toxic effects are often directed against certain microbes (antimicrobial effects). However, these compounds may also be toxic and directed against various host cells, especially when the number of eosinophils is elevated and thus the concentration of these compounds is high in local tissue sites, which may ultimately result in tissue damage and thus HES. **Neoplastic eosinophils triggered by various PDGFR mutant forms express and release increased amounts of certain cytokines, including OSM, when compared to normal eosinophils. Abbreviations: HE, hypereosinophilia; HES, HE syndrome; GM-CSF, granulocyte/macrophage colony-stimulating factor; IL, Interleukin; TGF, transforming growth factor; TNF, tumor necrosis factor; OSM, oncostatin M; SCF, stem cell factor; TLR-2, toll-like receptor-2; LFA, leukocyte function antigen; LTC_4_, leukotriene C_4_; PAF, platelet-activating factor; PGE, prostaglandin E; TXB_2_, thromboxane B_2_; PAI-2, plasminogen activator inhibitor-2.

Supplemental Table S3*

Primary Immunodeficiency Disorders (PID) Associated with (Hyper)Eosinophilia

-----------------------------------------------------------------------------------------------------------------

PID Involved Gene

Subtype/s Functional Defect Inheritance

-----------------------------------------------------------------------------------------------------------------

Combined Immunodeficiencies:

ADA Deficiency *ADA*  Elevated lymphotoxic AR

metabolites

ZAP70 deficiency** *ZAP70* Intracellular signaling AR

abnormality

CD3γ deficiency** *CD3G*  Altered T-cell receptor AR

expression in T cells

MHC II deficiency** *RFXANK* Impaired antigen AR

presentation by APCs

TCRα deficiency *TRAC* T cell receptor loss AR

MALT1 deficiency *MALT1*  Blocked NF-kB activation AR

Combined Immunodeficiencies with Associated Syndromes or Feature-Complex:

Omenn syndrome *RAG 1/2* Abnormal TCR generation AR

(OS)** *IL7RA*  Defect in IL-7RA AR

*IL*-*2RG* Defect in cytokine

receptor signaling AR

*CHD7* Altered chromatin AR

organization

*LIG4* DNA DSB repair defect AR

*ADA* Elevated lymphotoxic AR

metabolites

*RMRP* Mitochondrial RNA AR

processing defects

*CARD11* Altered TCR/BCR induced AR

NF-kB activation

*ARTEMIS* DNA repair defect AR

DiGeorge syndrome 22q11.2 Portions of chromosome 22 AD

deleted (gene candidates?)

Ataxia-telangiectasia *ATM* DNA break repair defect AR

Wiscott Aldrich *WAS* Actin cytoskeleton AR

syndrome (WAS) abnormality

Netherton syndrome *SPINK5* Pro-Th2 and stratum AR

(NS) corneum detachment

Hyper-IgE syndrome  *STAT3* Altered STAT signaling AD

(HIES)** *DOCK8* Defects in cytoskeletal AR

organization

TYK2 deficiency *TYK2* Altered cytokine- AR

(HIES-like) induced signaling

Predominantly Antibody Deficiencies:

Common variable unknown unknown variable

immunodeficiency

disorders (CVID)

CD40L deficiency *CD40L* Defects in Ig- XL

isotype switching

CD40 deficiency** *CD40* Defects in Ig- AR

isotype switching

Selective IgA unknown IgA deficiency unknown

deficiency

Other Diseases Associated with Immune Cell Dysregulation:

Immunodysregulation *FOXP3*  Abnormal function XL

polyendocrinopathy & of regulatory T cells

enteropathy X-linked

(IPEX)**

Autoimmune *TNFRSF6* Impaired FAS/FASL AD

lymphoproliferative (*FAS*) mediated apoptosis

syndrome (ALPS)** in lymphocytes

Roifman syndrome *RNU4ATAC* Disrupted minor AR

intron splicing

Congenital defects of phagocyte number or function or both:

Kostmann disease *HAX1* Apoptosis in myelocytes AR

Cyclic neutropenia *ELANE* Abnormal neutrophil AD

granule development

STAT1 deficiency** *STAT1* Abnormal cytokine- AD

induced STAT signaling

Papillon-Lefevre *FPR1*  Defective chemotaxis of AR

syndrome (LPS) phagocytes

X-linked chronic *CYBB* Neutrophil oxidative XL

granulomatous disease burst deficiency

Defects of innate immunity:

Anhidrotic ectodermal *NEMO* Blocked NEMO-induced XL

dysplasia-immune NF-κB activation

deficiency (EDA-ID)

CARD9 deficiency *CARD9* Selective defect in defense AR

against fungal infection

Autoinflammatory Disorders:

NOMID/CINCA *CIAS1* Defect in regulation of AD

inflammation and apoptosis

Blau syndrome *NOD2* NF-κB hyper-activation AD

with cytokine storm

-----------------------------------------------------------------------------------------------------------------

*The table has been reproduced with modifications form: Navabi and Upton, Allergy Asthma Clin Immunol. 2016;12:27.^6^ More information is provided in this review article as well as in Okamoto and Morio, Allergol Int. 2021;70(4):415-420.^7^ **In these conditions and syndromes, marked eosinophilia or even hypereosinophilia has been described as a recurrent feature. Abbreviations: AR, autosomal recessive; AD, autosomal dominant; XL, X-chromosome-linked; NOMID/CINCA, neonatal onset multisystem inflammatory disease (NOMID) or chronic infantile neurologic cutaneous and articular syndrome (CINCA).

Supplemental Table S4

Conditions and Disorders that can Cause Blood Hypereosinophilia (HE)

-----------------------------------------------------------------------------------------------------------------

Reactive Non-Neoplastic Conditions - Secondary/Reactive HE (HE_R_)*

Chronic infections: viral, bacterial, fungal (e.g., aspergillosis)

Parasitosis (e.g., helminth infections)

Infestations (e.g., scabies)

Allergic or toxic drug reactions

Intoxication: toxic oil syndrome, others

Allergic disorders, including atopic dermatitis and allergic asthma

Acute and chronic graft-versus-host disease

Autoimmune disorders – rheumatologic disorders (eGPA)

Chronic inflammatory disorders, including IBD (ulcerative colitis)

Lymphoid variant of hypereosinophilic syndrome (L-HES)

Myeloid/Lymphoid Neoplasms with Eosinophilia and Tyrosine Kinase Gene Fusions**

Hematopoietic neoplasms with eosinophilia and rearranged *PDGFRA*

Hematopoietic neoplasms with eosinophilia and rearranged *PDGFRB*

Hematopoietic neoplasms with eosinophilia and rearranged *FGFR1*

Hematopoietic neoplasms with eosinophilia and *PCM1::JAK2*

Hematopoietic neoplasms with eosinophilia and *JAK2* point mutations

Hematopoietic neoplasms with eosinophilia and *FLT3* mutations

Hematopoietic neoplasms with eosinophilia and *ABL1* fusion genes (except *BCR::*ABL1)

Other WHO-Defined Neoplasms Associated with HE (examples)**

Chronic eosinophilic leukemia (now a WHO-defined MPN subset)

Chronic myeloid leukemia (CML-eo) – *BCR*::*ABL1* positive

Myeloproliferative neoplasms (MPN) with HE (MPN-eo)

Systemic mastocytosis (SM) with HE (SM-eo)***

Myelodysplastic syndrome/neoplasm (MDS) with HE (MDS-eo)

MPN/MDS overlap syndromes with HE (MPN/MDS-eo; e.g., CMML-eo)

AML with *CBFB::MYH11* and eosinophilia (AML-eo)

Neoplastic Conditions with Secondary/Reactive HE (Paraneoplastic HE_R_)*

Solid tumors/cancers (lung, GI-tract, others)

Langerhans cell histiocytosis

Hodgkin´s disease

B- or T cell Non-Hodgkin lymphoma (NHL)

B- or T cell leukemia

-----------------------------------------------------------------------------------------------------------------

*In these patient groups, eosinophilia is considered to be caused by cytokines that promote the growth and accumulation of eosinophils and their precursor cells. **In these disorders, eosinophils are usually derived from the neoplastic clone (from clonal stem cells). ***Eosinophilia, including HE, develops relatively frequently in patients with advanced systemic mastocytosis (SM), such as aggressive SM (ASM), but may also occur in indolent SM (ISM) or smoldering SM (SSM). Abbreviations: eGPA, eosinophilic granulomatosis with polyangiitis; IBD, inflammatory bowel disease; CML, chronic myeloid leukemia; MDS, myelodysplastic syndrome; MPN, myeloproliferative neoplasm; CMML, chronic myelomonocytic leukemia; AML, acute myeloid leukemia; GI tract, gastrointestinal tract; NHL, Non Hodgkin lymphoma. The table has been reproduced with modifications form: Valent et al. J Allergy Clin Immunol. 2012;130(3):607-612.e9.^2^

Supplemental Table S5

Hematopoietic Neoplasms with Hypereosinophilia (HE): Comparison between WHO-based Definitions (2022 Update) and Updated ICOG-EO-Definitions for Morphologically-Defined Disease Variants, Including Acute and Chronic Eosinophilic Leukemias

*-----------------------------------------------------------------------------------------------------------------*

**I. Molecular Classification and Recurrent Molecular Defects (as per WHO)**

1. Myeloid/Lymphoid Neoplasms with Eosinophilia and Tyrosine Kinase Gene Fusions

(MLN-TK)*

a) *PDGFRA*-rearranged neoplasms

b) *PDGFRB*-rearranged neoplasms

c) *FGFR1*-rearranged neoplasms

d) *JAK2*-rearranged neoplasms

e) *FLT3*-rearranged neoplasms

d) *ETV6::ABL1*-rearranged neoplasms

e) Other fusion genes: *ETV6::FGFR2*; *ETV6::LYN*; *ETV6::NTRK3*;

*RANBP2::ALK*; *BCR::RET*; *FGFR1OP::RET* (see also Figure S1)

2. Chronic Eosinophilic Leukemia (CEL) now among myeloproliferative neoplasms, MPN.

As per WHO, MLN-TK must be excluded to diagnose CEL

3. Other WHO-defined Myeloid Neoplasms with HE (MN-eo):

a) *BCR::ABL1*+ chronic myeloid leukemia (CML-eo)

b) *JAK2* V617F+ MPN with HE (MPN-eo)

c) *KIT* D816V+ systemic mastocytosis with HE (SM-eo)

d) *CBFB*-fusion gene-related acute myeloid leukemia (AML-eo; often AML-M4-eo)

e) Myelodysplastic syndromes/neoplasms with HE (MDS-eo)

f) Other WHO-defined myeloid neoplasms with HE

**II. Histopathologic Classification** Proposed Proposed

**(as per ICOG-EO)*** Abbreviation Definition/Criteria

**---------------------------------------------------------------------------------------------------------**

A. Acute Eosinophilic Leukemia AEL Neoplastic HE** and

Eosinophils ≥30%*** and

Myeloblasts ≥20%***

B. Chronic Eosinophilic Leukemia CEL Neoplastic HE** and

Eosinophils ≥30%*** and

Myeloblasts <20%*** and no underlying stem cell-, myeloid

or lymphoid neoplasm found****

C. Other Myeloid Neoplasm (MN) MN-eo MN or stem cell neoplasm

or Stem Cell Neoplasm with HE: by WHO or FAB criteria and

MPN-eo, MDS-eo, SM-eo, .. HE, but eosinophils <30%

**-----------------------------------------------------------------------------------------------------------------**

*The 2022-updated WHO classification includes eosinophilia as a common and salient feature, but states that eosinophilia may be even absent in these patients: Khoury et al, Leukemia 2022;36(7);1703-1719; a similar classification has been proposed by the International Consensus Classification (ICC) for hematologic malignancies (ICC): Tzankov et al, Virchows Arch 2022, in press; by contrast, the ICOG-EO defines HE as a pre-requisite to diagnose an eosinophil neoplasm. **The neoplastic nature (clonality) of eosinophils is confirmed by molecular studies (myeloid neoplasm-related mutations detected by NGS with a VAF ≥3% as per ICOG-EO and ≥2% by WHO) and/or the presence of very immature (apparently leukemic) eosinophils – often in the context of an increase in myeloblasts. ***Values for eosinophils and myeloblasts refer to the bone marrow smear. In acute myeloid leukemia and eosinophilic leukemias, eosinophils may be quite immature and may escape conventional morphological identification. In these rare cases, it is extremely difficult to establish the correct diagnosis because robust diagnostic (immunophenotypic or molecular) markers are not available. ****To diagnose CEL, the following molecular defects must be excluded as primary reason for HE: *BCR::ABL1*, *CBFB*::*MYH11*, *JAK2* V617F, *KIT* D816V. Note however, that disease variants (more than one myeloid neoplasm) and mutants can coexist in one patient and also in one founder clone (subclone-formation in stem cell compartments). Therefore, the diagnosis of an accompanying CEL can still be established in a patient with *BCR::ABL1*^+^ CML or *KIT* D816V^+^ systemic mastocytosis, provided that i) the morphologic criteria outlined in this table are fulfilled and ii) additional molecular lesions typically found in CEL as per ICOG-EO proposal (e.g., fusion genes involving *PDGFRA* or *FGFR1*) are detected. The table has been reproduced with updates and modifications from: Valent et al. J Allergy Clin Immunol. 2012;130(3):607-612.e9. The WHO terminology is based on the 2022-updated WHO classification of myeloid neoplasms: Khoury et al, Leukemia 2022;36(7);1703-1719.

Supplementary Table S6

**Typical Examples of HE-induced Organ Damage Leading to the Diagnosis of HES***

-----------------------------------------------------------------------------------------------------------------

Cardiovascular System:

Deep vein thrombosis

Arterial occlusive disease

Myocardial infarction

Cardiac thromboembolism

Endomyocardial fibrosis

Pulmonary thromboembolism

Vasculitis including polyangiitis

Vascular Stroke

Pulmonary (Lung):

Dyspnea (decreased oxygen saturation)

Pulmonary infiltrations in imaging studies

Severe bronchial symptoms (asthma/cough)

Pleural effusion (recurrent or persistent)

Gastrointestinal (GI Tract):

GI tract symptoms (including symptoms listed below) leading to weight loss

Severe persistent abdominal pain

Chronic severe diarrhea

Recurrent (chronic) nausea and vomiting

Recurrent (chronic) severe dyspepsia/reflux

Dermatologic (Skin/Mucosa):

Blister formation (recurrent)

Ulcerations (persistent or recurrent)

Chronic severe eczema

Severe recurrent erythema

Severe recurrent edema/angioedema

Central and/or Peripheral Nervous System

Any persistent or recurrent neurologícal deficit

Paresis (hemiparesis, quadriparesis)

Persistent or recurrent mental impairment

Severe memory impairment

Local or multiple ischemic lesions in imaging studies

Brain cortical infarction (watershed infarction)**

-----------------------------------------------------------------------------------------------------------------

*Symptoms have been described as HE-induced (HE-related) in the available literature. The symptoms must be HE-related and have no other known etiology to count as feature (criteria) of HES. Most symptoms are recurrent and severe or even disabling. However, some of the pathologies are single events and still count as HES (examples: myocardial infarction or stroke). It is also worth noting that HE-induced organ damage may or may not be reversible – and both forms should qualify as HES if the organ damage is clinically relevant (e.g., persistent or recurrent). Sources: Kuang et al., J Allergy Clin Immunol Pract. 2020;8(8):2718-2726.e2; Lee and Ahn, J Neurol Sci. 2014;347(1-2):281-287; Requena et al., J Allergy Clin Immunol Pract. 2022;10(8):2125-2134; Leiferman and Peters, J Allergy Clin Immunol Pract 2018;6(5):1462-1482.e6. Abbreviations: HE, hypereosinophilia; HES, hypereosinophilic syndrome. **Brain cortical infarcts of watershed distribution have recently been described as HES manifestation in patients with neoplastic HE by Tennenbaum et al., Stroke. 2021;52(10):e605-e609.

Supplementary Table S7

**Organ-Restricted (Inflammatory) Conditions Accompanied by HE***

-----------------------------------------------------------------------------------------------------------------

Eosinophilic Gastrointestinal Disorders (EGID):

Eosinophilic esophagitis

Eosinophilic gastritis

Eosinophilic duodenitis

Eosinophilic gastroenteritis

Eosinophilic cholecystitis

Eosinophilic colitis

Eosinophilic pancreatitis

Eosinophilic hepatitis

Eosinophilic ascites

Pulmonary Eosinophilic Syndromes:

Eosinophilic asthma

Eosinophilic bronchitis

Eosinophilic pneumonia (acute and chronic)

Eosinophil pleuritis

Eosinophilic rhinosinusitis and nasal polyposis

Eosinophilic nephritis

Eosinophilic cystitis

Eosinophilic endometritis and myometritis

Eosinophilic oophoritis

Eosinophilic prostatitis

Eosinophilic mastitis

Eosinophilic Ocular Disorders:

Allergic conjunctivitis (seasonal and perenial)

Giant papillary conjunctivitis

Keratoconjunctivitis (atopic and vernal)

Eosinophilic myocarditis

Eosinophilic coronary periarteritis

Eosinophilic panniculitis

Eosinophilic synovitis

Eosinophilic fasciitis (Shulman’s syndrome)

Dermatological Conditions/Diseases**

-----------------------------------------------------------------------------------------------------------------

In addition to the disorders listed in this table, there are many other chronic conditions where blood or/and tissue HE is detectable, such as tissue fibrosis, ocular disorders, atopic dermatitis, nasal polyposis, interstitial nephritis, and acute necrotizing myocarditis. IgE, immunoglobulin E. *Depending on the histology, involvement pattern, severity and duration of symptoms and findings these organ-restricted conditions and syndromes may or may not fulfil the criteria of HES, which is then classified as reactive or idiopathic HES, depending on results obtained from additional investigations. **Dermatologic conditions including inflammatory reactions are listed in Supplementary Table S8.

Abbreviations: HE, hypereosinophilia; HES, hypereosinophilic syndrome.

Supplementary Table S8

**Dermatological Diseases Accompanied by Eosinophilia***

**-----------------------------------------------------------------------------------------------------------------**

I. Cutaneous diseases in which eosinophils are a principal feature

in histopathologic diagnoses:

Annular erythema of infancy

Eosinophilic, polymorphic, and pruritic eruption associated with radiotherapy

Eosinophilic pustular folliculitis

Classical (Ofugi disease)

Infantile/neonatal

Human immunodeficiency virus (HIV)-associated

Eosinophilic annular erythema

Eosinophilic cellulitis (Wells syndrome)

Eosinophilic dermatosis of hematologic malignancy

Erythema toxicum neonatorum

Eosinophilic ulcer of oral mucosa

Eosinophilic vasculitis

Hypereosinophilic dermatitis of Nir-Westfried

Hypereosinophilic syndromes (HES)

IgG4-related cutaneous diseases**

Angiolymphoid hyperplasia with eosinophilia

Granuloma faciale

Kimura disease

Juvenile temporal arteritis

Pachydermatous eosinophilic dermatitis

II. Dermatologic diseases commonly associated with tissue eosinophils:

Arthropod bite and sting reactions

Autoantibody-associated blistering diseases

Pemphigoid

Pemphigus

Epidermolysis bullosa acquisita

Dermatoses of pregnancy

Pemphigoid gestationis

Polymorphic eruption of pregnancy, pruritic urticarial papules and plaques of pregnancy

Drug reactions

DRESS (drug reaction with eosinophilia and systemic symptoms) also known as drug-

induced hypersensitivity syndrome

Interstitial granulomatous drug reaction

Cutaneous involvement

Eosinophilic granulomatosis with polyangiitis (Churg-Strauss syndrome)

Nodules, eosinophilia, rheumatism, dermatitis, and swelling (NERDS)

Hereditary syndromes

Incontinentia pigmenti

Hyper IgE syndromes and other immunodeficiency syndromes

Autoimmune lymphoproliferative syndrome

Histiocytic diseases

Langerhans cell histiocytosis

Juvenile xanthogranuloma

Itchy, red bump disease (papular dermatitis)

Oid-oid disease (exudative discoid and lichenoid chronic dermatosis of

Sulzberger and Garbe)

Papuloerythroderma of Ofugi

Parasitic diseases/infestations

Cysticercosis, dirofilariasis, fascioliasis, gnathostomiasis, larva migrans, loiasis,

myiasis, onchocerciasis, paragonimiasis, schistosomiasis, strongyloidiasis, tungiasis

Scabies, pediculosis, pthiriasis, cimicosis

Swimmer’s itch (cercarial dermatitis) and seabather’s itch

Pruritic papular eruption of human immunodeficiency virus disease

Sclerosis or fibrosis

Eosinophilic fasciitis

Eosinophilia myalgia and toxic oil syndromes

Drugs including statins

Iron infusion

Lymphoma and leukemia

Graft versus host disease after hematopoietic stem cell transplantation

Urticaria and angioedema

Vasculitis

III. Eosinophils of limited or unreliable value in histopathologic diagnosis:

Granuloma annulare

Interstitial granulomatous dermatitis

Lymphoproliferative disorders excepting HES variants

Mycosis fungoides and Sézary syndrome

Peripheral T cell lymphoma

Anaplastic large cell lymphoma (except neutrophil-rich and eosinophil-rich variants)

Lymphomatoid papulosis

Other Neoplasms

Mastocytosis

Melanoma

Keratoacanthoma-type squamous cell carcinoma

Invasive squamous cell carcinoma

**-----------------------------------------------------------------------------------------------------------------**

*Modified from: Leiferman and Peters, J Allergy Clin Immunol Pract. 2018;6(5):1462-1482.e6.

**IgG4-related disease is a recently recognized clinical entity characterized by swelling or mass lesion(s), increased serum IgG4 levels, increased serum IgE, along with PB eosinophilia and tissue HE. The pathogenesis is thought to derive from cross-talk between innate and acquired immunity. Criteria for the diagnosis of IgG4-RD are principally histopathologic and include dense lymphoplasmacytic infiltrates, storiform fibrosis, eosinophil infiltration, and obliterative phlebitis. IgG4-producing plasma cells are found in many inflammatory disorders, but a ratio of IgG4/IgG-positive cells greater than 40% or absolute number of IgG4-positive cells per high-power field greater than 10 supports the diagnosis. Disorders now considered to be in the IgG4-RD spectrum have in common a strong association with eosinophils and fibrosis. In several of these patients, (tissue) HE is documented and the disease resembles (reactive) HES: Moussiegt et al., Autoimmun Rev. 2021;20(9):102889. Abbreviations: HES, hypereosinophilic syndrome.

**Legend to Figure S1**

Mutational landscape and cytokine (growth factor) receptor-induced signaling pathways driving eosinophilia and eosinophil activation in eosinophil neoplasms and reactive states.

Eosinophils develop normally in the bone marrow, mainly under the influence of certain cytokines or growth factors, including mainly interleukin-5 (IL-5), IL-3, and granulocyte/macrophage colony-stimulating factor (GM-CSF). These cytokines are released upon T cell-, mast cell-, and/or stroma cell activation, sometimes in conjunction with antigen-presenting cells via MHC class II presentation. The scheme provides an overview on mutated (rearranged) driver genes that act as transforming events associated with hematologic neoplasms and concomitant eosinophilia. Hyperactivating mutations of tyrosine kinases activate several critical downstream signaling cascades, with subsequent opening of chromatin and transcriptional reprogramming through e.g. STAT molecules which finally leads to neoplastic outgrowth of myeloid cells and eosinophils. Neoplastic eosinophilia is found in various hematologic (stem cell and myeloid) neoplasms presenting with a variety of different somatic mutations, predominantly in ´oncogenic´ tyrosine kinases and related fusion gene products. The orange box shows a compilation of most frequently identified fusion gene products in stem cell and myeloid neoplasms (that may be accompanied by eosinophilia). Moreover, a large number of mutations in various signaling molecules have also been associated with neoplastic conditions in eosinophilia (yellow stars). The mutated onco-proteins initiate various signal transduction cascades and thereby contribute to disease evolution and/or progression. Cytokine signaling is maintained through the common β chain of IL-5-, GM-CSF- and IL-3 receptors, which binds to Janus kinase 2 (JAK2). Mutational events promote hyperactive tyrosine kinase activity that evokes subsequently high levels of STAT1, a potential tumor suppressor, and STAT3, which is mutated in hyper-IgE syndrome. Hyper-activating STAT5B^N642H^ mutations promote enhanced STAT5 tyrosine phosphorylation. STAT3/5 activation is oncogenic and this leads to higher cytokine/growth factor sensitivity. An important interaction of STAT family members is illustrated with the glucocorticoid receptor (GR) that potentiates gene transcription via STAT protein interaction. The GR is e.g. N-domain bound to STAT5 acting as a transcriptional cofactor, but it can also repress or transcriptionally regulate inflammatory genes such as cytokines independently. Corticosteroids indeed exert multiple functionally relevant anti-inflammatory effects on T cells and eosinophils. Nuclear shuttling and efficient transformation through STAT3/5 action also requires RAS-RAF-MAPK and PI3K-AKT-mTOR signaling boosting GTPase signaling through RhoA/RAC-ROCK pathways. STAT5 signaling is also interwoven with mTOR activation and phosphorylation of STAT5, and docking of STAT5 to GAB scaffold proteins that trigger PI3K-AKT-mTOR signaling. High pYSTAT5 levels can form STAT oligomers involving also STAT1/3/5 oligomerisation. DNA looping indicates high oncogene transcription to promote eosinophil cell survival, proliferation, activation and release of toxic substances that can cause organ/tissue damage. Furthermore, metabolic events, adhesion and migration are regulated by eosinopoietic cytokines and downstream signaling pathways. The expression of negative regulators such as the SOCS proteins or of E3-ubiquitin ligases (that degrade STATs or other key molecules) or of tumor suppressor protein interaction such as TP53 interplay with STATs are also under JAK-STAT pathway patrol or interwoven. However, negative regulator transcriptional loci can be methylated and their expression is often low or lost, also due to genetic deletion or mutation, at the end insufficient to block hyperactive JAK-STAT signaling or lost capacity to bind to hyperactive tyrosine kinases. Loss-of-function mutations in the critical tumor suppressor protein TP53 are also detected in hematopoietic neoplasms associated with eosinophilia. Furthermore, various epigenetic-modifier proteins are found to be mutated in such neoplasms, several known to interact with STAT1/3/5, TP53 or GR transcription factors, including histone methyltransferase protein of polycomb repressive complex 2 (PRC2) EZH2, methylcytosine dioxygenase TET2, SET binding protein 1 (SETBP1), isocitrate dehydrogenase 2 (IDH2), polycomb group protein Additional Sex Comb Like 1 (ASXL1) and pre-mRNA-splicing factor 3b subunit 1 (SF3B). Furthermore, acetyl transferases such as CREB-binding protein (CBP) or the E1A-binding protein P300 (p300) are essential to facilitate transcription. Therapeutic agents known to target key oncogenic proteins and signaling pathways in hematologic disorders associated with eosinophilia are shown in black boxes.

Abbreviations: GEF, guanine exchange factor; SOS, son of sevenless; GTP, guanine triphosphate; mTOR, mechanistic target of rapamycin; JAK, Janus kinase; STAT, signal transducer and activator of transcription; SOCS, suppressor of cytokine signaling; CBL, E3 ubiquitin-protein ligase CBL-C; MDM2, murine double minute 2; MDMX, murine double minute X; TP53, tumor suppressor protein 53; BCL-2, B cell lymphoma 2; MCL-1, myeloid cell leukemia 1; MAPK1/2, mitogen-activated protein kinase 1/2; MEK, mitogen-activated protein kinase kinase; PI3K, phosphatidylinositol-4,5-bisphosphate-3-kinase.
