# Supplementary material for: Proposed refined diagnostic criteria and classification of eosinophil disorders and related syndromes
Source: Allergy. 2022 Oct 19;78(1):47–59. doi: 10.1111/all.15544 (PMC9797433; doi:10.1111/all.15544)
Supplement: Supplementary file 2 — Figure S1 [file ALL-78-47-s002.pdf]

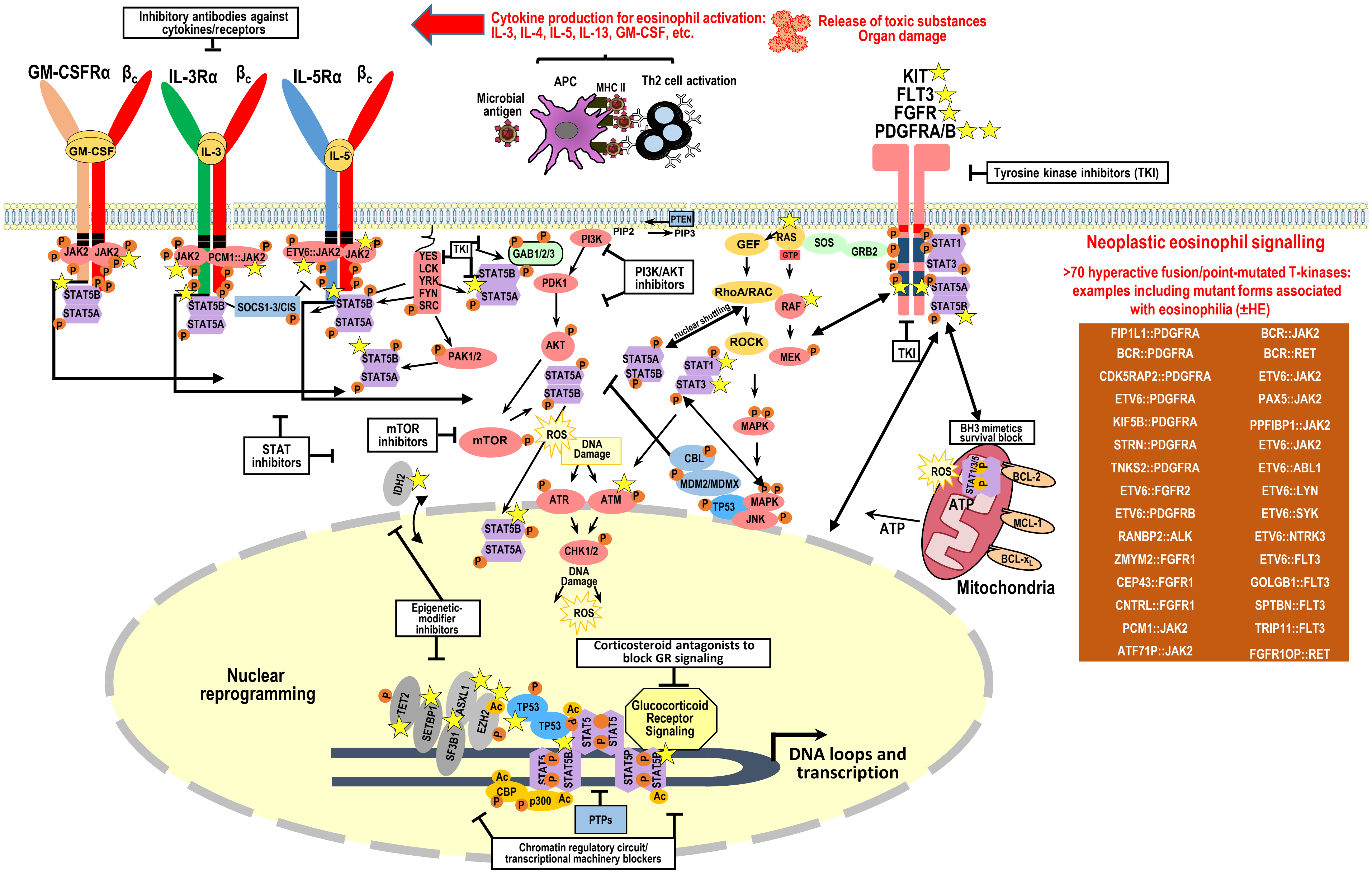

Valent et al, Supplemental Figure S1

Legend to Figure S1

Mutational landscape and cytokine (growth factor) receptor-induced signaling pathways driving eosinophilia and eosinophil activation in eosinophil neoplasms and reactive states.

Eosinophils develop normally in the bone marrow, mainly under the influence of certain cytokines or growth factors, including mainly interleukin-5 (IL-5), IL-3, and granulocyte/macrophage colony-stimulating factor (GM-CSF). These cytokines are released upon T cell-, mast cell-, and/or stroma cell activation, sometimes in conjunction with antigen-presenting cells via MHC class II presentation. In certain myeloid neoplasms, hyperactivating mutations of tyrosine kinases activate several critical downstream signaling cascades, with subsequent opening of chromatin and transcriptional reprogramming through e.g. STAT molecules which finally leads to neoplastic outgrowth of myeloid cells and eosinophils. Neoplastic eosinophilia is found in various stem cell and myeloid neoplasms presenting with a variety of different somatic mutations, predominantly in ´oncogenic´ tyrosine kinases and related fusion gene products. The orange box shows a compilation of most frequently identified fusion gene products that may be accompanied by eosinophilia. Moreover, a number of mutations in various signaling molecules have also been associated with neoplastic eosinophilia (yellow stars). The mutated oncoproteins initiate various signal transduction cascades and thereby contribute to disease evolution and/or progression. Cytokine signaling is maintained through the common β chain (shown in red color) of IL-5-, GM-CSF- and IL-3 receptors, which binds to Janus kinase 2 (JAK2). Mutational events promote hyperactive tyrosine kinase activity that evokes subsequently high levels of STAT1, a potential tumor suppressor, and STAT3, which is mutated in hyper-IgE syndrome. Hyper-activating STAT5BN642H mutations promote enhanced STAT5 tyrosine phosphorylation. STAT3/5 activation is oncogenic and this leads to higher cytokine/growth factor sensitivity. An important interaction of STAT family members is illustrated with the glucocorticoid receptor (GR) that potentiates gene transcription via STAT protein interaction. The GR is e.g. N-domain bound to STAT5 acting as a transcriptional cofactor, but it can also repress or transcriptionally regulate inflammatory genes such as cytokines independently. Corticosteroids indeed exert multiple functionally relevant anti-inflammatory effects on T cells and eosinophils. Nuclear shuttling and efficient transformation through STAT3/5 action also requires RAS-RAF-MAPK and PI3K-AKT-mTOR signaling boosting GTPase signaling through RhoA/RAC-ROCK pathways. STAT5 signaling is also interwoven with mTOR activation and phosphorylation of STAT5, and docking of STAT5 to GAB scaffold proteins that trigger PI3K-AKT-mTOR signaling. High pYSTAT5 levels can form STAT oligomers involving also STAT1/3/5 oligomerisation. DNA looping indicates high oncogene transcription to promote eosinophil cell survival, proliferation, activation and release of toxic substances that can cause organ/tissue damage. Furthermore, metabolic events, adhesion and migration are regulated by eosinopoietic cytokines and downstream signaling pathways. The expression of negative regulators such as the SOCS proteins or of E3-ubiquitin ligases (that degrade STATs or other key molecules) or of tumor suppressor protein interaction such as TP53 interplay with STATs are also under JAK-STAT pathway patrol or interwoven. However, negative regulator transcriptional loci can be methylated and their expression is often low or lost, also due to genetic deletion or mutation, at the end insufficient to block hyperactive JAK-STAT signaling or lost capacity to bind to hyperactive tyrosine kinases. Loss-of-function mutations in the critical tumor suppressor protein TP53 are also detected in hematopoietic neoplasms associated with eosinophilia. Furthermore, various epigenetic-modifier proteins are found to be mutated in such neoplasms, several known to interact with STAT1/3/5, TP53 or GR transcription factors, including histone methyltransferase protein of polycomb repressive complex 2 (PRC2) EZH2, methylcytosine dioxygenase TET2, SET binding protein 1 (SETBP1), isocitrate dehydrogenase 2 (IDH2), polycomb group protein Additional Sex Comb Like 1 (ASXL1) and pre-mRNA-splicing factor 3b subunit 1 (SF3B). Furthermore, acetyl transferases such as CREB-binding protein (CBP) or the E1A-binding protein P300 (p300) are essential to facilitate transcription. Therapeutic agents known to target key oncogenic proteins and signaling pathways in hematologic disorders associated with eosinophilia are shown in black boxes. Abbreviations: GEF, guanine exchange factor; SOS, son of sevenless; GTP, guanine triphosphate; mTOR, mechanistic target of rapamycin; JAK, Janus kinase; STAT, signal transducer and activator of transcription; SOCS, suppressor of cytokine signaling; CBL, E3 ubiquitin-protein ligase CBL-C; MDM2, murine double minute 2; MDMX, murine double minute X; TP53, tumor suppressor protein 53; BCL-2, B cell lymphoma 2; MCL-1, myeloid cell leukemia 1; MAPK1/2, mitogen-activated protein kinase 1/2; MEK, mitogen-activated protein kinase kinase; PI3K, phosphatidylinositol-4,5-bisphosphate-3-kinase.
